# Atorvastatin Effectively Inhibits Ancestral and Two Emerging Variants of SARS-CoV-2 *in vitro*

**DOI:** 10.3389/fmicb.2022.721103

**Published:** 2022-03-18

**Authors:** María I. Zapata-Cardona, Lizdany Flórez-Álvarez, Wildeman Zapata-Builes, Ariadna L. Guerra-Sandoval, Carlos M. Guerra-Almonacid, Jaime Hincapié-García, María T. Rugeles, Juan C. Hernandez

**Affiliations:** ^1^Grupo Inmunovirología, Facultad de Medicina, Universidad de Antioquia UdeA, Medellín, Colombia; ^2^Grupo Infettare, Facultad de Medicina, Universidad Cooperativa de Colombia, Medellín, Colombia; ^3^Grupo de investigación GIRYSOUT, Universidad del Tolima, Ibagué, Colombia; ^4^Grupo de investigación, Promoción y prevención farmacéutica, Facultad de ciencias farmacéuticas y alimentarias, Universidad de Antioquia UdeA, Medellín, Colombia

**Keywords:** atorvastatin, SARS-CoV-2, antiviral, molecular docking, COVID-19, variants

## Abstract

This article evaluated the *in vitro* antiviral effect of atorvastatin (ATV) against SARS-CoV-2 and identified the interaction affinity between this compound and two SARS-CoV-2 proteins. The antiviral activity of atorvastatin against this virus was evaluated by three different treatment strategies [(i) pre-post treatment, (ii) pre-infection treatment, and (iii) post-infection treatment] using Vero E6 and Caco-2 cells. The interaction of atorvastatin with RdRp (RNA-dependent RNA polymerase) and 3CL protease (3-chymotrypsin-like protease) was evaluated by molecular docking. The CC50s (half-maximal cytotoxic concentrations) obtained for ATV were 50.3 and 64.5 μM in Vero E6 and Caco-2, respectively. This compound showed antiviral activity against SARS-CoV-2 D614G strain in Vero E6 with median effective concentrations (EC50s) of 15.4, 12.1, and 11.1 μM by pre-post, pre-infection, and post-infection treatments, respectively. ATV also inhibited Delta and Mu variants by pre-post treatment (EC50s of 16.8 and 21.1 μM, respectively). In addition, ATV showed an antiviral effect against the D614G strain independent of the cell line (EC50 of 7.4 μM in Caco-2). The interaction of atorvastatin with SARS-CoV-2 RdRp and 3CL protease yielded a binding affinity of −6.7 kcal/mol and −7.5 kcal/mol, respectively. Our study demonstrated the *in vitro* antiviral activity of atorvastatin against the ancestral SARS-CoV-2 D614G strain and two emerging variants (Delta and Mu), with an independent effect of the cell line. A favorable binding affinity between ATV and viral proteins by bioinformatics methods was found. Due to the extensive clinical experience of atorvastatin use, it could prove valuable in the treatment of COVID-19.

## Introduction

COVID-19 (coronavirus disease 2019) is a disease caused by the SARS-CoV-2 virus (Severe Acute Respiratory Syndrome Coronavirus 2), reported for the first time in Wuhan, China (Lotfi et al., 2020). On March 11, 2020, the WHO (World Health Organization) declared COVID-19 a pandemic (WHO, 2020a). Since then, it has affected 221 countries and territories worldwide, causing enormous human health consequences (Lotfi et al., 2020; WHO, 2020b).

SARS-CoV-2 is an enveloped virus. Its viral particle has 80–160 nm in diameter and is characterized by the presence of Spike (S) protein homotrimers that protrude from the viral envelope, a helical nucleocapsid, and a single-stranded positive-sense RNA [ssRNA (+)], of approximately 30 kb in size that is translated into 16 non-structural proteins (NSPs), four structural, and nine accessory proteins, each one with important participation in the viral replicative cycle (Blaess et al., 2020; Kumar et al., 2020; Mendonça et al., 2020; Simabuco et al., 2021; V’kovski et al., 2021).

Currently, COVID-19 disease control has been based on symptom management, including the use of convalescent plasma, synthetic antibodies, interferon, low-dose corticosteroids, IL-1 and IL-16 inhibitors, Remdesivir, Baricitinib, Lopinavir/Ritonavir, and in severe cases, supportive care (oxygen and mechanical ventilation; Esakandari et al., 2020; Singh et al., 2020). Although there are some approved drugs (Boopathi et al., 2020; FDA, 2020; Pan et al., 2021), there is still no conclusive information on their effectiveness. For instance, Remdesivir, a nucleotide analog prodrug that inhibits the activity of SARS-CoV-2 RdRp, was approved by FDA (Food and Drug Administration) for the treatment of COVID-19 in adults and certain pediatric patients who require hospitalization (Ader et al., 2021; FDA, 2021a). However, several trials have found no clinical benefits or faster viral clearance associated with the use of this drug in severe COVID-19 patients (Wang Y. et al., 2020; Ader et al., 2021). On the other hand, the FDA approved the combination of remdesivir with an anti-inflammatory agent (baricitinib) to improve the clinical results of monotherapy (FDA, 2021b). This combination managed to reduce recovery time and accelerate improvement in clinical status among COVID-19 patients (Kalil et al., 2020); however, it is only approved in hospitalized patients requiring supplemental oxygen, mechanical ventilation, or extracorporeal membrane oxygenation (FDA, 2021b).

Considering the above, it is necessary to continue with the search for effective and specific anti-SARS-CoV-2 antivirals (Sacco et al., 2020). The development of new drugs involves the evaluation of pharmacokinetics and pharmacodynamic safety, with the implementation of large-scale production and distribution, a process that takes many years. Due to this panorama and the rapid expansion of the pandemic, the repurposing of clinically approved drugs may represent a useful COVID-19 treatment option in terms of safety, cost-effectiveness, and timeliness (Yuan et al., 2020).

Atorvastatin (ATV) belongs to statins, a group of hypolipidemic drugs that inhibit HMG-CoA reductase. ATV was approved by the FDA and EMA (European Medicines Agency) to prevent cardiovascular events in patients with cardiac risk factors or with abnormal lipid profiles (PFIZER, 2010; McIver and Siddique, 2020). The ability of ATV to modulate cholesterol synthesis has previously been related to antiviral activity against viruses such as Hepatitis C virus (HCV; Kim et al., 2007), Dengue virus (DENV; Villareal et al., 2015), Zika virus (ZIKV; Españo et al., 2019), Influenza A virus (IAV; Mehrbod et al., 2012), and human parainfluenza virus type 1 (HPIV-1; Bajimaya et al., 2017). This drug also modulates several cellular metabolic pathways that could affect the viral replicative cycle (Feng et al., 2011; Villareal et al., 2015).

It has been proposed that statins could be an effective therapeutic strategy against SARS-CoV-2 infection (Castiglione et al., 2020). Recently, it has been reported that statin treatment was associated with a reduced risk of mortality in patients diagnosed with COVID-19 (Tan et al., 2020; Wang et al., 2021). However, there is no *in vitro* evidence of its antiviral effect against SARS-CoV-2. This article evaluated the *in vitro* antiviral effect of the ATV against the D614G strain, Delta, and Mu variants of SARS-CoV-2, and identified the interaction affinity between ATV and two viral proteins, using an *in silico* structure-based molecular docking approach.

## Materials and Methods

### Preparation of Compounds

ATV was purchased from Biogen Idec, Inc. (Cambridge, MA). It was solubilized in dimethyl sulfoxide (DMSO; Sigma-Aldrich) at a final concentration of 100 mM. For *in vitro* assays, ATV was used at concentrations of 0.98–250 μM, in which its biological activity has been reported (Isusi et al., 2000; Parquet et al., 2010; Jones et al., 2017; Shrivastava-Ranjan et al., 2018). Chloroquine (CQ) was bought from Sigma-Aldrich (St. Louis, MO, United States), and it was diluted to 15 mM in phosphate-buffered saline (PBS, Lonza, Rockland, ME, United States). CQ concentrations (6.3–100 μM) were selected according to previous studies (Uzunova et al., 2020). Heparin was bought from B. Braun Melsungen AG and was diluted to 1 mg/ml in PBS. Heparin was used at concentrations from 6.3 to 100 μg/ml (Mycroft-West et al., 2020). ATV and CQ stock solutions were frozen at −80°C, and Heparin was stored at 4°C, until use.

### Cells and Virus

Vero E6 cells from *Cercopithecus aethiops* kidney were donated by Instituto Nacional de Salud, Bogotá-Colombia (Dr. José Usme, 11 April 2020), and human colon carcinoma Caco-2 cells (HTB37) were purchased from the American Type Culture Collection (ATCC, Manassas, VA, United States). Cells were grown in Dulbecco’s Modified Eagle Medium (DMEM, Sigma-Aldrich) supplemented with 2% heat-inactivated fetal bovine serum (FBS, Gibco), 2 mM L-glutamine (Gibco), and 1% Penicillin–Streptomycin (Gibco). The incubation conditions were 37°C, 5% CO_2_ atmosphere, and relative humidity. The cells were infected with viral stocks produced from three Colombian isolates of SARS-CoV-2: D614G strain (EPI_ISL_536399; Díaz et al., 2020), Delta (EPI_ISL_5103929), and Mu (EPI_ISL_4005445) variants. Vero E6 cells were used to produce the viral stocks. All experimental studies involving infectious SARS-CoV-2 were conducted within a biosafety level 3 laboratory (BSL3), according to the conditions set out in Biosafety in Microbiological and Biomedical Laboratories (Meechan and Potts, 2020).

### Cytotoxicity Assay

Cytotoxicity of ATV was assessed using the MTT (3-(4,5-dimethylthiazol-2-yl)-2,5-diphenyltetrazolium bromide) assay. Briefly, Vero E6 and Caco-2 cells were seeded in 96-well plates at a density of 1.0 × 10^4^ and 2.5 × 10^4^ cells/well, respectively. Cultures were incubated for 24 h at 37°C and 5% CO_2_. Then, double serial dilutions of ATV ranging from 0.98 to 250 μM were prepared and added to each well. After 48 h of incubation, the supernatants were removed, cells were washed twice with PBS, and an MTT solution (2 mg/ml) was added. Plates were incubated for 2 h at 37°C, with 5% CO_2,_ protected from light. After, DMSO was added to each well to solubilize the formazan crystals. Optical density (OD) was recorded at 570 nm using a Multiskan GO spectrophotometer (Thermo). Cell viability was calculated based on the OD of the untreated controls compared with treated cells. Concentrations that maintained more than 80% of cell viability after treatment were considered nontoxic and were used for the antiviral evaluation. Chloroquine (from 6.3 to 100 μM) and Heparin (from 6.3 to 100 μg/ml) were used as positive inhibition controls. For the MTT assay, two independent experiments with four replicates each were performed (*n* = 8).

### Evaluation of the Antiviral Activity

The antiviral activity of ATV against SARS-CoV-2 was evaluated initially through a *pre-post treatment* in Vero E6 cells. Briefly, cells were seeded in 96-well plates at a density of 1.0 × 10^4^ cells/well. Cells were incubated for 24 h, at 37°C, with 5% CO_2_, and then pretreated with double dilutions of ATV (3.9–31.2 μM) for 1 h before infection. Treatment was then removed, and cells were infected with SARS-CoV-2 stock at an MOI (multiplicity of infection) of 0.01 in DMEM with 2% FBS. Cultures were incubated for 1 h at 37°C. The inoculum was removed and replaced newly by the same pre-treatment dilutions of ATV. After 48 h of treatment, the cell culture supernatants were harvested and stored at −80°C to be quantified by plaque assay. The supernatant of infected untreated cells was used as the infection control.

Additionally, two antiviral strategies were done: *pre-infection treatment* (ATV was added 1 h before infection and was removed before viral infection) and *post-infection treatment* (ATV was added after infection). The viral titer in cell culture supernatants was quantified by plaque assay for both strategies. For post-infection treatment, viral RNA in Vero E6 monolayers was also quantified by real-time RT-PCR. Chloroquine (Uzunova et al., 2020; from 12.5 to 100 μM) and Heparin (Mycroft-West et al., 2020; Tandon et al., 2021; 12.5–100 μg/ml) were included as positive inhibition controls. Two independent experiments with four replicates per experiment were performed (*n* = 8).

The anti-SARS-CoV-2 activity of ATV (0.98–7.8 μM) was also evaluated in the Caco-2 cell line using the *pre-post treatment* strategy. Cells (2.5 × 10^4^ cells/well) were infected with D614G strain at an MOI of 0.01 in DMEM with 2% FBS. Chloroquine was used as positive inhibition control (50 μM). The infectious viral particles in supernatants were quantified by plaque assay.

### Viral Quantification by Plaque Assay

The reduction of SARS-CoV-2 titer in cell supernatants was quantified by plaque assay (Mendoza et al., 2020; Yepes-Perez et al., 2021). Briefly, tenfold serial dilutions of the supernatants obtained from the antiviral assay were prepared in DMEM with 2% FBS and used to inoculate confluent monolayers of Vero E6 cells into plates of 24 wells (1.1 × 10^5^ cells/well). After 1 h of incubation, the viral inoculum was removed, and cells were overlaid with 1.5% carboxymethyl-cellulose in DMEM with 2% FBS. Then, the cultures were incubated for 4 days at 37°C, with 5% CO_2_. After incubation, the monolayers were washed twice with PBS, fixed with 4% Formaldehyde, and plaques were revealed with 1% Crystal violet solution (Sigma-Aldrich). Plaques were counted and used to calculate the number of plaque-forming units per milliliter (PFU/ml). The reduction in the viral titer after treatment compared to the infection control (untreated infected cells) was expressed as an inhibition percentage. Two independent experiments with two replicates per each were performed (*n* = 4).

### Viral Quantification by Real-Time RT-PCR

Real-time RT-PCR quantified the reduction of viral RNA in Vero E6 monolayers treated by post-infection treatment. Viral RNA extraction was carried out from monolayers using the Quick-RNA™ Viral Kit (Zymo Research), following the manufacturer’s instructions. SARS-CoV-2 viral RNA was quantified using the Luna® Universal Probe One-Step RT-qPCR Kit (New England Biolabs, MA, United States). The reaction included the oligos and probe for the E gene and the conditions reported in the Berlin real-time RT-PCR protocol (Corman et al., 2020) with a modification, according to One-Step RT-qPCR Kit manufacturer recommendations in a reverse transcription (55°C for 18 min) and alignment/extension step (60°C for 30 s). The RT-PCR reactions were carried out in a CFX-96 Bio-Rad thermal cycler (Bio-Rad, CA, United States). Two independent experiments in duplicate were performed for each strategy.

The number of viral RNA copies was calculated by extrapolating the cycle at which viral stock or its dilutions cross the fluorescence threshold (Ct) in a standard curve constructed with a serially diluted 3,180 bp plasmid containing the E gene in a concentration of 2 × 10^9^ copies/μl, gently donated by Dr. Jaime Castellanos from Universidad del Bosque (Bogotá, Colombia).

### Molecular Docking

The molecular docking simulation was used to determine the binding affinity between atorvastatin with two SARS-CoV-2 proteins involved in the viral replication. The selected proteins were considered and obtained from the Protein Data Bank (PDB; Berman et al., 2000). RdRp (PDB: 6M71; Gao et al., 2020) and 3CL protease (PDB: 6M2N; Su et al., 2020) crystals from SARS-CoV-2 were selected. The protein structures were subjected to preparation at Discovery Studio (BIOVIA, 2020) and AutoDock Tools (ADT). The ligands were drawn and optimized using Avogadro software (Hanwell et al., 2012) and ADT.

PrankWeb (Jendele et al., 2019) was used to check the binding site coordinate and specify the amino acids in the SARS-CoV-2 proteins and pockets. Protein plus (Schöning-Stierand et al., 2020) was used to determine the size, shape, and functional group descriptors of the selected pockets. Couplings were carried out using Auto Dock vina version 4.2.6 (Trott and Olson, 2010), configuring a box with dimensions x: 24 Å, y: 24 Å, z: 24 Å for each simulation and using exhaustiveness of 20. The grid box coordinates were defined as follows: x: 116.7829 Å, y: 109.9570 Å, z: 123.9430 Å for RdRp (PDB:6 M71), and x: −47.585 Å, y: 1.135 Å, z: −5.600 Å for 3CL protease (PDB:6M2N; Table 1).

**Table 1:** PrankWeb result summary of RdRp and 3CL protease of SARS-CoV-2.

Target protein	Amino acids make up the pocket	Depth (Å)	Surface (Å^2^)	Volume (Å^3^)	Hydrophobicity ratio	Amino acid composition: (apolar, polar, positive, negative amino acid) ratio	Grid center (Å)		
							x	Y	z
RdRp (PDB:6 M71)	TRP 617, ASP 618, TYR 619, LEU 758, SER 759, ASP 760, ASP 761, ALA 762, LYS 798, TRP 800, GLU 811, CYS 813, SER 814	14.62	1223.18	839.19	0.54	0.45, 0.33, 0.12, 0.09	116.7829	109.9570	123.9430
3CL protease (PDB:6M2N)	PHE 140, LEU 141, ASN 142, GLY143, SER144, CYS 145, HIS163, HIS 164, MET 165, GLU 166, ASP 187, GLN 189, THR 25, THR 26, LEU 27, HIS 41, CYS 44, THR 45, MET 49.	17.18	762.59	633.10	0.41	0.37, 0.37, 0.17, 0.10	−47.5857	1.1355	−5.6005

Remdesivir (Koulgi et al., 2020; Kokic et al., 2021) and chloroquine (Li et al., 2020; Nimgampalle et al., 2020) were used as positive controls of RdRp and 3CL protease interactions, respectively.

### Statistical Analysis

All data were analyzed with GraphPad Prism (La Jolla, CA, United States) and presented as mean ± SEM (standard error of the mean). Statistical differences were evaluated *via* Student’s *t*-test or Mann–Whitney U test, according to the normality of the data. A value of *p* ≤ 0.05 was considered significant. The EC50 values represent the concentration of each medicament that reduces viral titer by 50%. The CC50 values represent the concentration that causes 50% toxicity on Vero E6. The corresponding dose–response curves were fitted by non-linear regression analysis using a sigmoidal model. The calculated selectivity index (SI) represents the ratio of CC50 to EC50. The viral RNA copies of the treated Vero E6 monolayers were calculated by interpolating from the standard curve in a linear regression analysis.

## Results

### Atorvastatin Did Not Affect Cell Viability of Vero E6 and Caco-2 Cells

**Figure 1:**
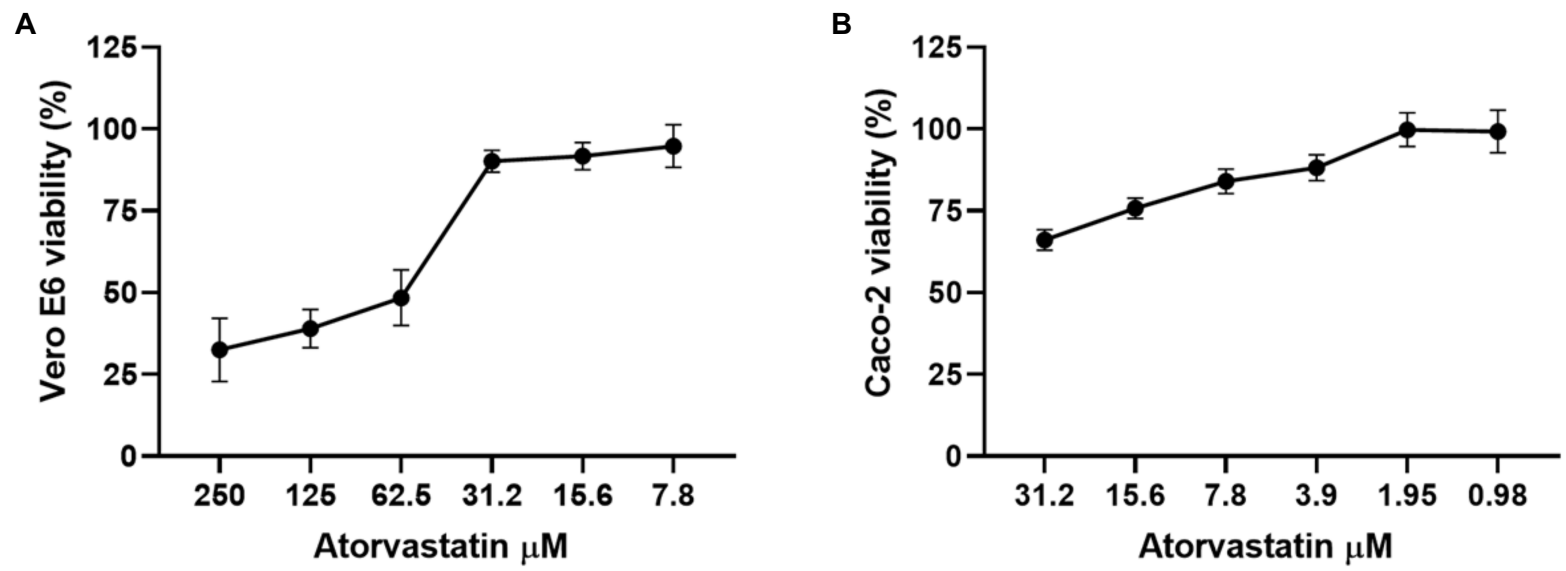
Atorvastatin did not affect the cell viability of Vero E6 and Caco-2. Viability of Vero E6 **(A)** and Caco-2 **(B)** after 48 h of atorvastatin treatment. Data were presented as Mean ± SEM. The viability percentages of the treated cell were calculated based on untreated control. Two independent experiments with four replicates each were performed (*n* = 8).

Before the antiviral activity evaluation, the cytotoxic effect of ATV was determined by MTT assay. As shown in Figure 1A, Vero E6 viability was higher than 90% at 31.2 μM or lower ATV concentrations. In contrast, viability decreased drastically when the highest concentrations (62.5 to 250 μM) were assessed. On the other hand, Caco-2 cells treated with ATV showed viability percentages higher than 84% at 7.8 μM or lower ATV concentrations (Figure 1B). The CC50s calculated for ATV were 50.3 and 64.5 μM in Vero E6 and Caco-2 cells, respectively.

Vero E6 viability was not affected by CQ and Heparin treatments (positive inhibition controls) at any of the evaluated concentrations (Marín-Palma et al., 2021). The CC50s obtained for CQ and Heparin were more than 100 μM and 100 μg/ml, respectively, in Vero E6 cells. In addition, CQ at 50 μM did not affect the viability of Caco-2 cells.

### ATV Exhibited Antiviral Effects Against SARS-CoV-2 D614G Strain in a Dose-Dependent Manner

**Figure 2:**
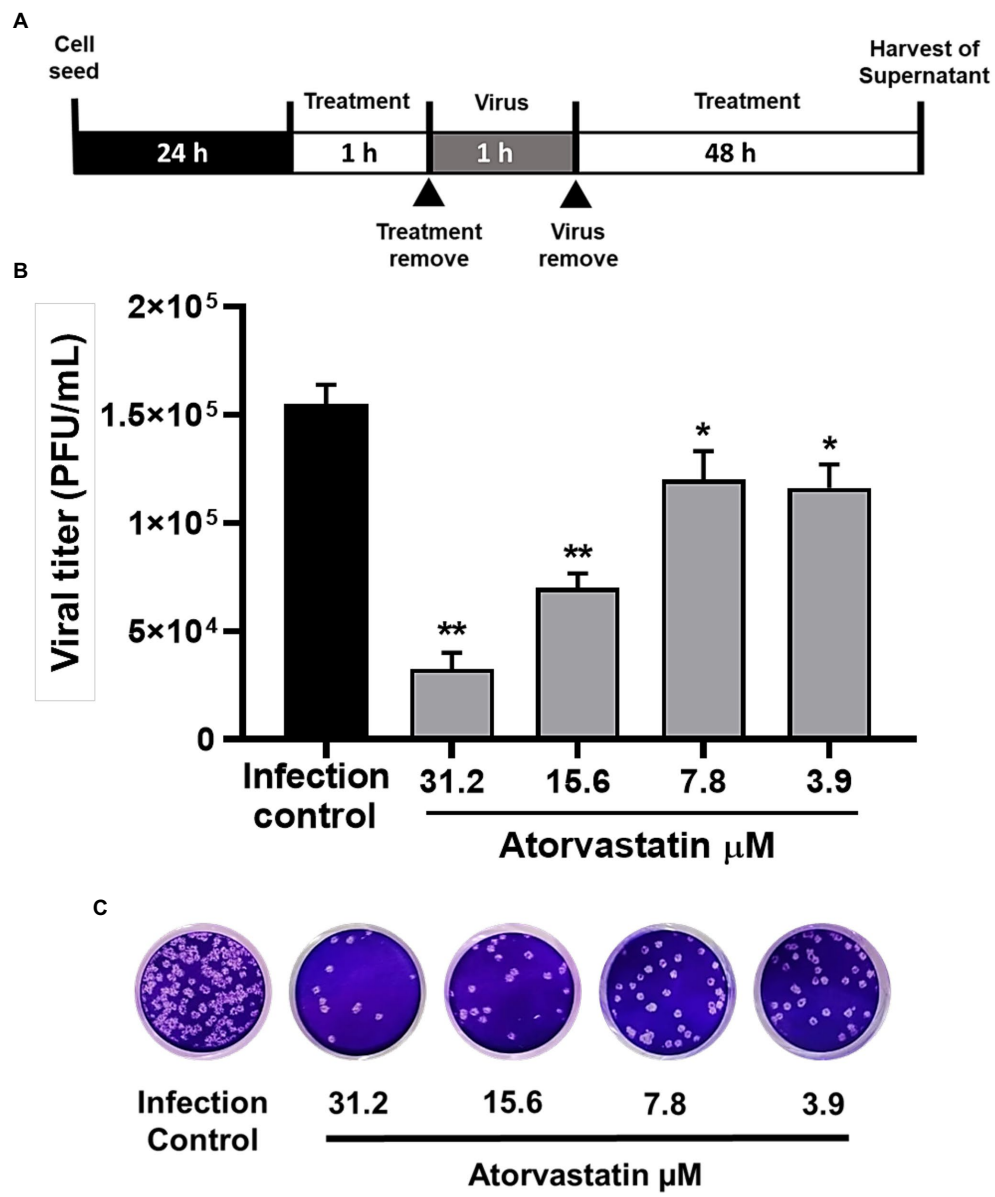
Atorvastatin exhibited an antiviral effect against the SARS-CoV-2 D614G strain in a dose-dependent manner. **(A)** Schematic representation of the pre-post treatment strategy. **(B)** Reduction of the D614G strain titer (PFU/ml) in Vero E6 supernatants after pre-post treatment with ATV (*n* = 4). Data were presented as Mean ± SEM. Mann–Whitney test ^*^p ≤ 0.05 and ^**^p ≤ 0.01 **(C)** Representative plaques of each treatment condition are shown.

To evaluate the antiviral activity of ATV against the SARS-CoV-2 D614G strain, a pre-post treatment strategy was performed on Vero E6 cells. ATV showed inhibition percentages for D614G strain of 79% (*p* = 0.002), 54.8% (*p* = 0.002), 22.6% (*p* = 0.04), and 25% (*p* = 0.03) at 31.2, 15.6, 7.8, and 3.9 μM concentrations, respectively (Figure 2). Based on these data, the EC50 calculated for ATV was 15.4 μM in Vero E6, with a selectivity index of 3.3 (Table 2).

**Table 2:** CC50, EC50, and SI values for ATV in Vero E6 and Caco-2 cells infected with SARS-CoV-2.

Compound	Cell line	CC_50_ (μM)	Virus	Treatment strategy	EC50 (μM)	IS
Atorvastatin	Vero E6	50.3	D614G strain	Pre-post treatment	15.4	3.3
				Pre-infection treatment	12.1	4.2
				Post-infection treatment	11.1	4.5
			Delta variant	Pre-post treatment	16.8	3.0
			Mu variant	Pre-post treatment	21.1	2.4
	Caco-2	64.5	D614G strain	Pre-post treatment	7.4	8.7

Chloroquine (positive inhibition control) exhibited antiviral activity against D614G strain at 100 μM (100%, *p* = 0.002), 50 μM (99.9%, *p* = 0.002), 25 μM (97.5%, *p* = 0.002), and 12.5 μM (55.7%, *p* = 0.002; Supplementary Figure 1A). An EC50 value of 13.5 μM was calculated, with a selectivity index of >7.4.

### ATV Inhibited D614G Strain Through Post-infection Treatment

**Figure 3:**
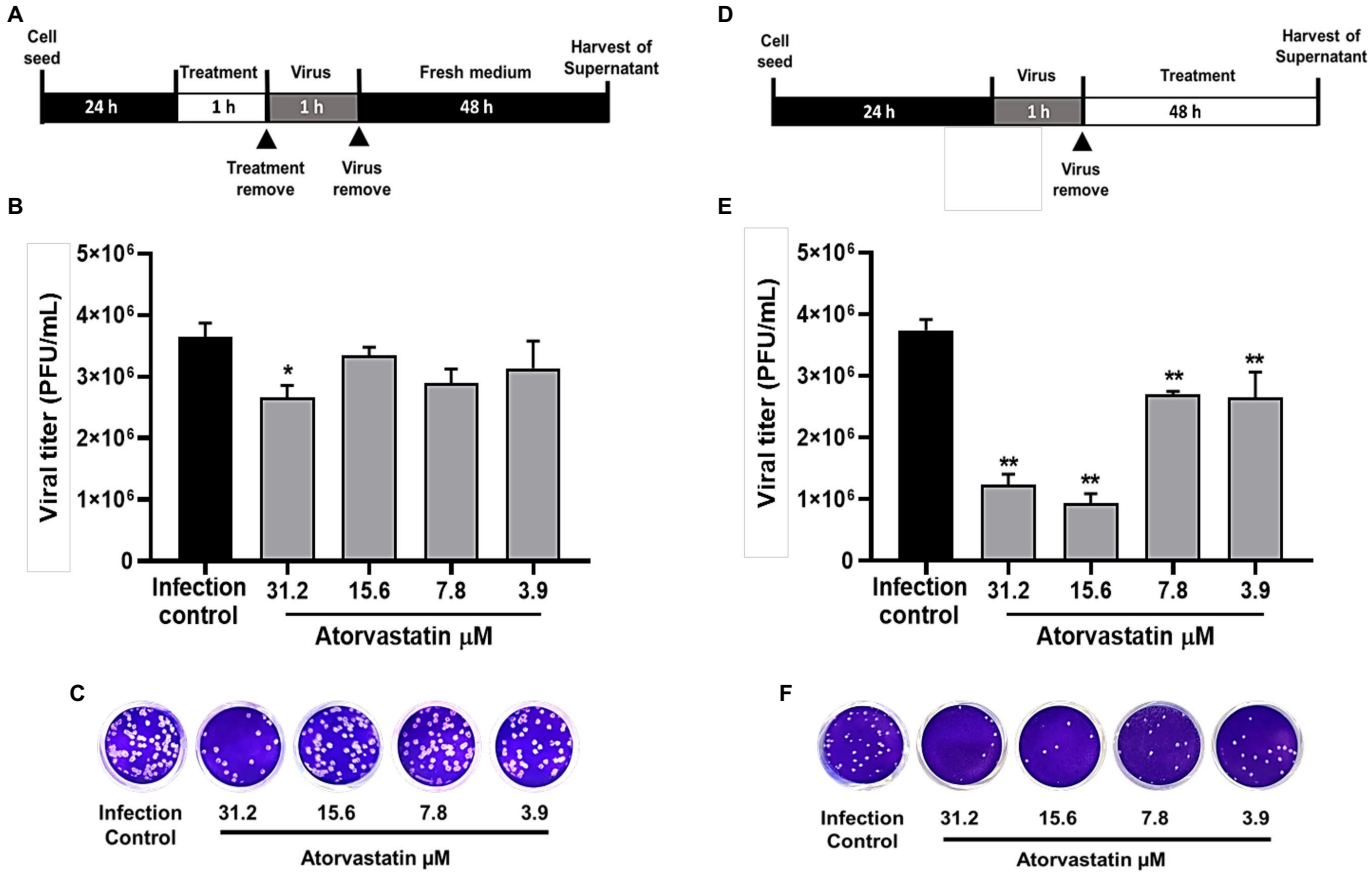
ATV inhibited D614G strain during pre-infection and post-infection treatment. **(A)** Schematic representation of the pre-infection treatment. **(B)** Reduction of the D614G strain titer (PFU/ml) in Vero E6 supernatants after pre-infection treatment with ATV. Data were presented as Mean ± SEM (*n* = 4). Mann–Whitney test ^*^p ≤ 0.05 and ^**^p ≤ 0.01. **(C)** Representative plaques of pre-infection treatment against the D614G strain are shown. **(D)** Schematic representation of the post-infection treatment. **(E)** Reduction of D614G strain titer (PFU/ml) in Vero E6 supernatants after post-infection treatment with ATV. **(F)** Representative images of plaques formed in each treatment condition are shown.

Once an antiviral effect against the SARS-CoV-2 D614G strain was observed by pre-post treatment in Vero E6, the pre-infection and post-infection treatments were done separately to infer the step of the viral replicative cycle affected by the ATV treatment. The anti-SARS-CoV-2 activity in pre-infection treatment was observed at 31.2 μM (inhibition of 26.9%, *p* = 0.012; Figures 3A–C). The EC50 calculated for ATV was 12.1 μM, with a selectivity index of 4.2, through this treatment strategy (Table 2). In comparison, as shown in Figures 3D–F, the viral titer of D614G strain was significantly reduced through post-infection treatment with ATV at all evaluated concentrations. Inhibition percentages of 66.9% (*p* = 0.004), 75% (*p* = 0.004), 27.9% (*p* = 0.004), and 29.2% (*p* = 0.006) were obtained at ATV concentrations of 31.2, 15.6, 7.8, and 3.9 μM, respectively (EC50 = 11.1 μM, SI = 4.5).

Heparin and Chloroquine were the positive inhibition controls during pre-infection and post-infection treatments, respectively. By pre-infection treatment, Heparin inhibited SARS-CoV-2 D614G strain at 100 μg/ml (80.6%, *p* = 0.028), 50 μg/ml (82.9%, *p* = 0.028), 25 μg/ml (87.3%, *p* = 0.028), and 12.5 μg/ml (79.9%, *p* = 0.028; Supplementary Figure 1B). On the other hand, as shown in Supplementary Figure 1C, CQ significantly inhibited D614G strain at 100, 50, 25, and 12.5 μM, with inhibition percentages of 99.2% (*p* = 0.009), 98.3% (*p* = 0.009), 74.8% (*p* = 0.009), and 23.6% (*p* = 0.033), respectively, by post-infection treatment (EC50 = 12.8, SI > 7.8).

**Figure 4:**
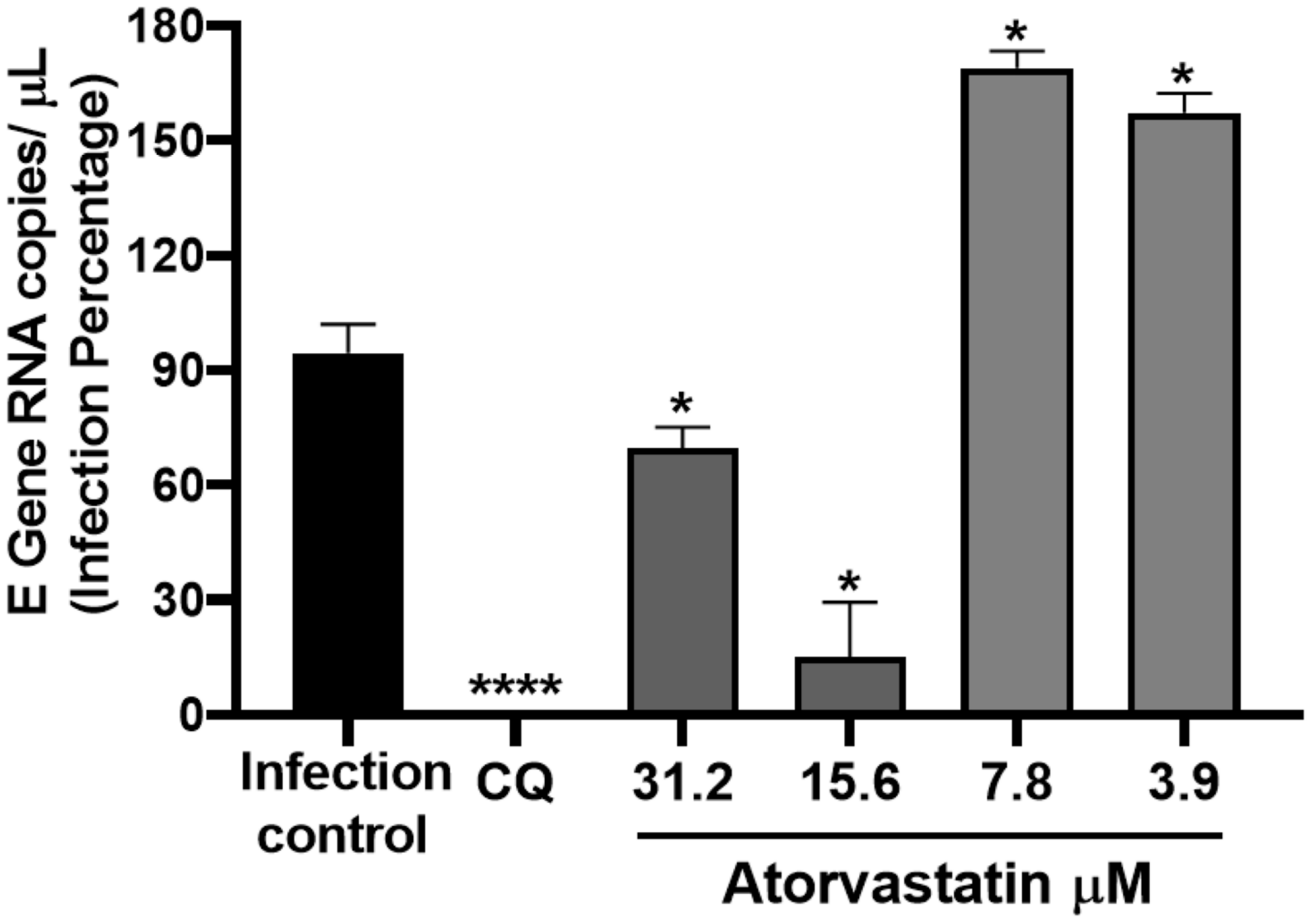
ATV affected SARS-CoV-2 D614G replication. Infection percentage obtained of the quantification of SARS-CoV-2 RNA (E Gene RNA copies/μl) in Vero E6 monolayers after post-infection treatment with ATV. CQ was used as a positive control of viral RNA inhibition (*n* = 4). Student’s *t*-test or Mann–Whitney U test ^*^p ≤ 0.05 and ^****^p < 0.0001.

Once it was demonstrated that ATV inhibits the number of infectious viral particles released by post-infection treatment in Vero E6, intracellular viral RNA was quantified to determine if this compound affects the viral replication stage. ATV inhibited the D614G strain RNA at 31.2 μM (30.17%, *p* = 0.03) and 15.6 μM (84.8%, *p* = 0.03; Figure 4). An inhibition of 99% (*p* < 0.0001) was obtained with CQ (positive control of RNA inhibition).

### The Antiviral Effect of Atorvastatin Was Independent of the SARS-CoV-2 Variant

**Figure 5:**
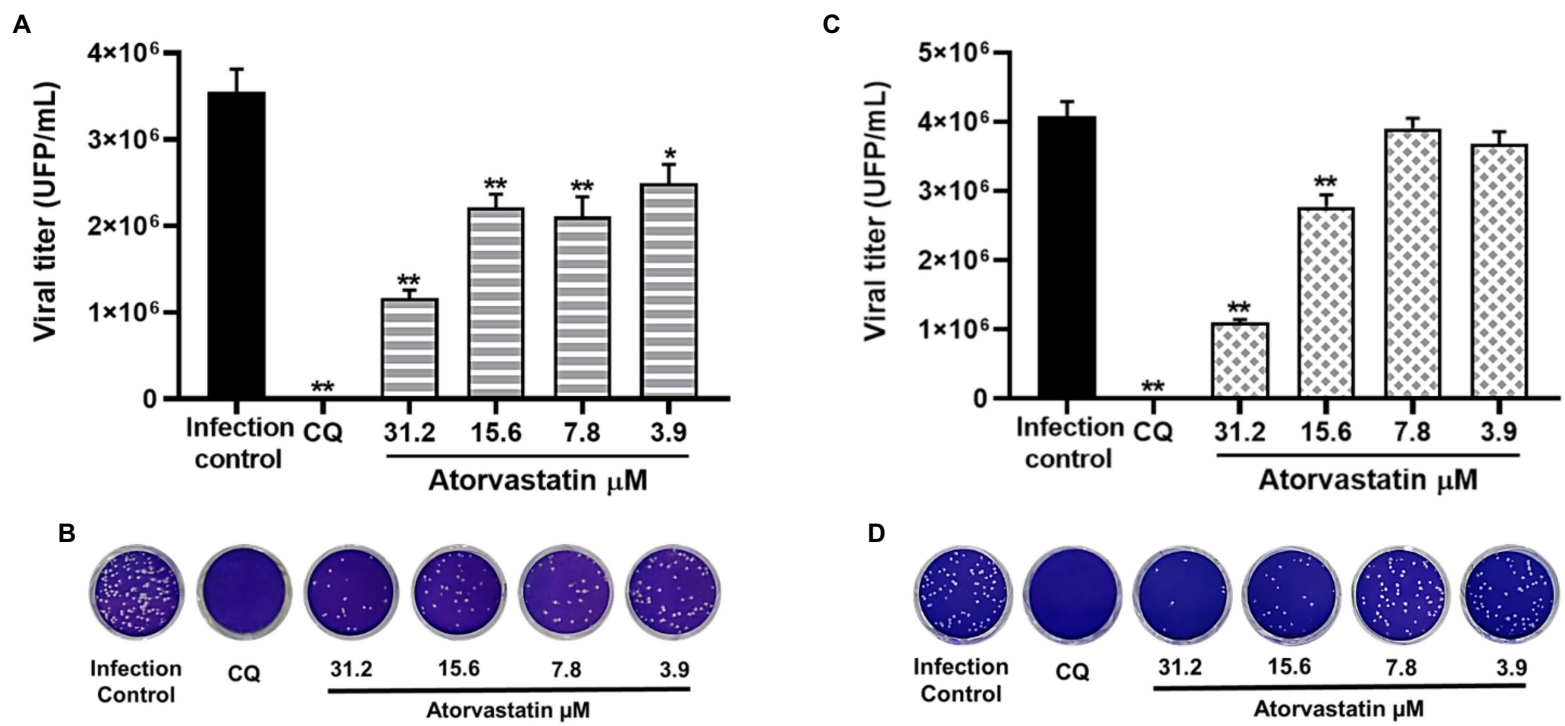
The antiviral effect of Atorvastatin was independent of the infecting SARS-CoV-2 variant. **(A)** Reduction of Delta variant titer (PFU/ml) in Vero E6 supernatants after pre-post treatment with ATV (*n* = 4). **(B)** Representative plaques of ATV treatment against the SARS-CoV-2 Delta variant are shown. **(C)** Reduction of Mu variant titer (PFU/ml) in Vero E6 supernatants after pre-post treatment with ATV (*n* = 4). **(D)** Representative plaques of ATV treatment against the SARS-CoV-2 Mu variant are shown. Chloroquine (CQ) was used as a positive inhibition control. Data were presented as Mean ± SEM. Mann–Whitney test ^*^p ≤ 0.05 and ^**^p ≤ 0.01.

Considering that ATV showed inhibition against the D614G strain, this compound was evaluated against the SARS-CoV-2 Delta and Mu variants in Vero E6 cells. ATV exhibited antiviral activity against Delta variant at 31.2 μM, 15.6, 7.8, and 3.9 μM concentrations, with inhibition percentages of 67.1% (*p* = 0.002), 37.6% (*p* = 0.002), 40.6% (*p* = 0.002), and 29.8% (*p* = 0.013), respectively, by pre-post treatment (Figures 5A,B). The EC50 value calculated for ATV against Delta was 16.8 μM, with a SI of 3 (Table 2). As shown in Figures 5C,D, ATV inhibited Mu variant at 31.2 μM (73.1%, *p* = 0.002) and 15.6 μM (32.2%, *p* = 0.002) using pre-post treatment. The EC50 for ATV against Mu was 21.1 μM, with a SI of 2.4 (Table 2). These results indicated that the anti-SARS-CoV-2 effect of ATV was independent of the infecting variant. Chloroquine showed antiviral activity against the Delta (99.99%, *p* = 0.002) and Mu (100%, *p* = 0.002) variants in Vero E6, by pre-post treatment (Figure 5).

### Atorvastatin Inhibited SARS-CoV-2 in a Human Cell Line

**Figure 6:**
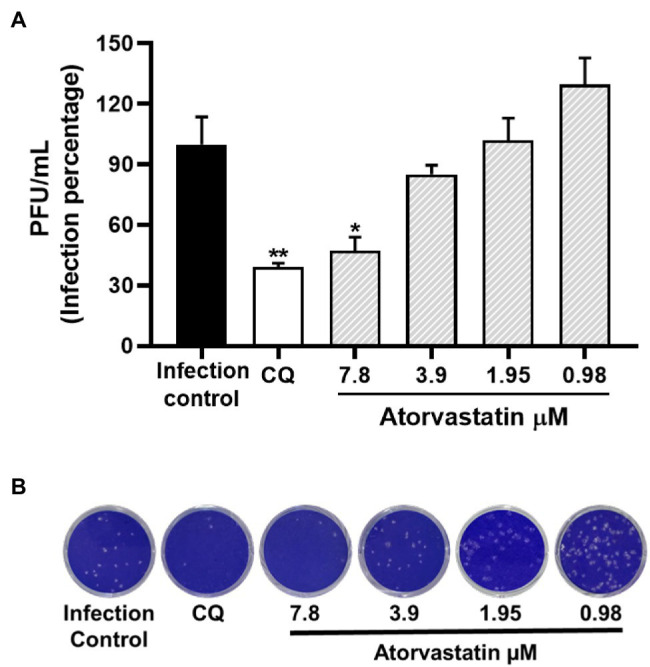
Atorvastatin inhibited SARS-CoV-2 in a human cell line. **(A)** Reduction of D614G strain titer (PFU/ml) in Caco-2 cells supernatants after pre-post treatment with ATV (*n* = 4). **(B)** Representative plaques of ATV treatment against the SARS-CoV-2 D614G are shown. Chloroquine (CQ) was used as a positive inhibition control. Data were presented as Mean ± SEM. Mann–Whitney test ^*^p ≤ 0.05 and ^**^p ≤ 0.01.

Because ATV showed anti-SARS-CoV-2 activity in Vero E6, the antiviral effect of this compound was evaluated using the human epithelial cell line Caco-2. As shown in Figure 6, ATV inhibited significantly the SARS-CoV-2 D614G strain in Caco-2 at 7.8 μM (52.7%, *p* = 0.03), by pre-post treatment (EC50 = 7.4 μM, SI = 8.7). Further, CQ (50 μM) inhibited SARS-CoV-2 in Caco-2 cells (60.9%, *p* = 0.004) using a pre-post treatment strategy (Figure 6).

### Binding Site Determination of Viral Proteins

Before molecular docking, pockets and expected amino acids were selected to assess the interaction affinity of two SARS-CoV-2 proteins with ATV. SARS-CoV-2 RdRp (PDB:6 M71) had 37 pockets. The pocket with the best score was number one (value of 8.79). It consisted of 23 amino acids, a solvent accessible surface (SAS) of 88, and a surface area conformed with 49 atoms. This pocket was selected because it included the active site of the enzyme (SER 759, ASP 760, and ASP761; Ahmad et al., 2020). On the other hand, the 3CL protease (PDB:6M2N) showed 15 pockets. The highest score was obtained in the pocket number one, with a value of 12.65. It consisted of 19 amino acids, the solvent accessible surface (SAS) of 86, and a surface area conformed with 50 atoms. This pocket included the active catalytic-domain amino acids (CYS 145 and HIS41) of SARS-CoV-2 3CL protease (Alexpandi et al., 2020). Chemical properties, size description, and the grid centers of these pocket sites were shown in Table 1.

### ATV Demonstrated Favorable Binding Affinities With SARS-CoV-2 Proteins by Molecular Docking

The binding affinity of atorvastatin revealed a high interaction affinity and coupling with the different hydrophobic pockets selected from the RdRp (−6.7 kcal/mol) and 3CL protease (−7.5 kcal/mol) of SARS-CoV-2 (Table 3).

**Table 3:** Molecular docking of atorvastatin and positive controls of interaction (Remdesivir and Chloroquine) with two SARS-CoV-2 proteins.

Ligand	Score with the pocket of viral proteins (kcal/mol)	
	RdRp (PDB:6 M71)	3CL protease (PDB:6M2N)
Atorvastatin	−6.7	−7.5
Remdesivir	−7.1	-
Chloroquine	-	−6.3

**Figure 7:**
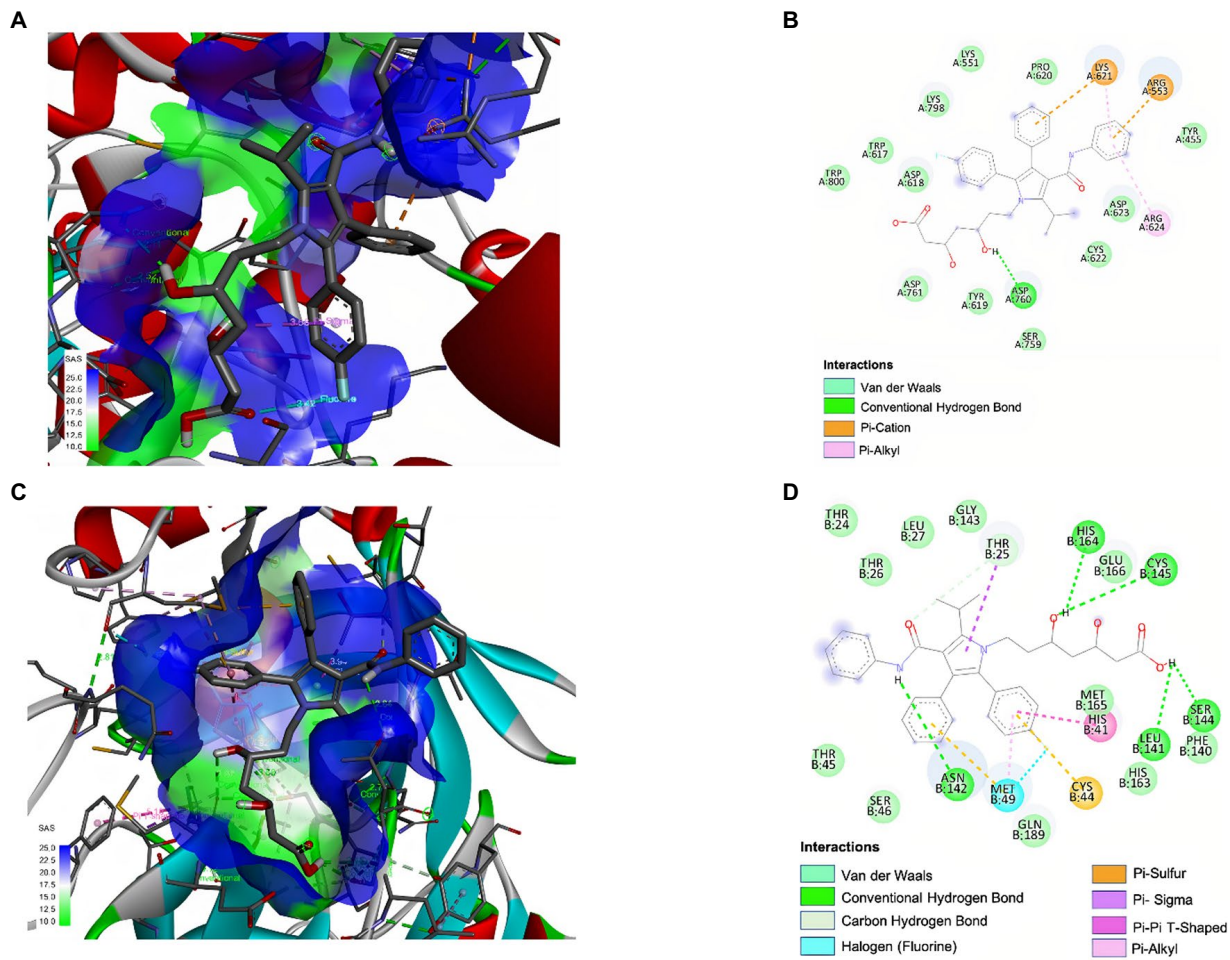
Interaction of ATV with SARS-CoV-2 RdRp and 3CL protease by molecular docking. 3D and 2D representations of the main interaction of ATV and two SARS-CoV-2 proteins by molecular docking. The images represent ATV interaction with: RdRp (PDB:6 M71) depicted in 3D **(A)** and 2D **(B)**, and 3CL protease (PDB: 6M2N) of SARS-CoV-2 depicted in 3D **(C)** and 2D **(D)**. The interactions formed in the complexes are described in each figure. The images were obtained using BIOVIA Discovery Studio Visualizer 16.1.

The molecular interactions of ATV with RdRp and 3CL protease of SARS-CoV-2 were numerous and explained the high affinity presented (Figure 7). Specifically, ATV formed three conventional hydrogen bonds with the amino acids ARG 624 and one with ASP 760 of SARS-CoV-2 RdRp (distances of 3.88 Å, 2.52 Å, 3.16 Å, and 2.11 Å, respectively). Other types of interactions, such as hydrophobic and electrostatic interactions, were found. ATV formed π-alkyl and π-cation bonds with ARG 624 and LYS 621 of RdRp, respectively (Figures 7A,B). On the other hand, ATV formed six hydrogen bonds with HIS 164, CYS 145, ASN 142, LEU 141, SER 144, and THR 25 of 3CL protease, with distances of 3.12 Å, 3.20 Å, 2.64 Å, 2.48 Å, 3.02 Å, and 2.94 Å, respectively. In this complex, it also participated hydrophobic interactions such as π-sigma, π-alkyl, and π-Sulfur (Figures 7C,D).

Remdesivir and Chloroquine (positive controls of interaction) showed a high affinity with RdRp (−7.1 kcal/mol) and 3CL protease (−6.3 kcal/mol), respectively. Remdesivir and RdRp formed 11 hydrogen bonds with ASP 623, ASP 623, ARG 553, LYS 798, LYS 621, LYS 621, CYS 622, CYS 622, ASP761, ASP 761, and PRO 620 (distances of 3.88, 3.10, 3.27, 3.27, 3.38, 2.92, 3.33, 3.40, 2.89, 2.91, and 3.39 Å, respectively). Other types of interactions, such as hydrophobic and electrostatic interactions, were found in this pocket (Supplementary Figures 2A–D). On the other hand, Chloroquine and 3CL protease formed 13 hydrogen bonds with CYS 44, HIS 164, HIS 164, HIS 164, HIS 164, HIS 164, HIS 164, HIS 163, HIS 163, HIS 163, HIS 163, HIS 163, and MET 49 (distances of 3.20 Å, 2.98 Å, 3.12 Å, 2.91 Å, 2.96 Å, 3.00 Å, 3.24 Å, 3.10 Å, 2.91 Å,3.16 Å, 3.54 Å, 3.04 Å, and 2.81 Å, respectively). Further, hydrophobic interactions such as π-alkyl, π-Sigma, and π-π T-shaped were found (Supplementary Figures 2C,D).

## Discussion

In the present study, the antiviral effect of atorvastatin against SARS-CoV-2 D614G strain (Korber et al., 2020; Khateeb et al., 2021) was identified by the treatment of Vero E6 cells at different times of infection. ATV has been shown to inhibit HMG-CoA reductase, affecting cholesterol synthesis and the production of isoprenoid metabolites (Españo et al., 2019). Previously, it has been reported that cholesterol-modifying drugs could exert antiviral effects by modulating cellular metabolic pathways required for the replicative cycle or by direct effect against viral particles (Sanders et al., 2020; Schmidt et al., 2020). Concerning SARS-CoV-2, it has been suggested that statins may help to reduce viral entry and transmission (Tan et al., 2020). Based on our results, ATV treatment seems to affect different stages of the SARS-CoV-2 replicative cycle, possibly, adhesion (Wang et al., 2021), endocytosis (Lu et al., 2008; Minz et al., 2020), fusion (Wang et al., 2021), replication (Wolff et al., 2020), or viral particle assembly (Klein et al., 2020; Mukherjee et al., 2020), where cholesterol is involved.

A previous study reported that another statin, Fluvastatin, reduced SARS-CoV-2 entry into the respiratory epithelium cells (Zapatero-Belinchón et al., 2021). Similar to this report, our study demonstrated that ATV inhibited D614G strain at 31.2 μM by pretreating cells for 1 h. These findings suggest that ATV treatment affects the early stages of the viral replicative cycle. This effect could be due to the fact that the ACE2 cell receptors are located in lipid rafts, cholesterol-rich microdomains present at the cell membrane (Lu et al., 2008; Minz et al., 2020). Wang et al. showed that cholesterol transports ACE2 to sites where SARS-CoV-2 effectively enters the cell (Wang et al., 2021). Therefore, an alteration at the cholesterol level present at the cell membrane by ATV treatment could affect viral attachment; however, additional experiments are needed to confirm this mechanism.

It has also been reported that statins upregulate ACE2 expression through epigenetic modifications (Fedson et al., 2020). Although this would be expected to promote viral infection, there is no current evidence indicating that statins enhance viral entry into host cells (Al-Horani et al., 2020). Conversely, the increase in the ACE2 receptor expression could be associated with a reduction of the severity of acute respiratory distress syndrome (ARDS; Wösten-van Asperen et al., 2013). In this regard, ACE2 metabolizes the vasoconstrictor peptide angiotensin II to produce angiotensin 1–7, which reduces inflammation, tissue damage, and pulmonary edema (González-Rayasa et al., 2020). Ongoing clinical studies would provide information on the effectiveness of ATV in preventing or mitigating these effects in patients with COVID-19 (ClinicalTrials.gov, 2020).

In this study, Vero E6 cells were treated with ATV for 48 h after infection, obtaining a reduction of the infectious SARS-CoV-2 D614G particles at all evaluated concentrations. Regarding this strategy, it has been reported that statins could affect steps, such as replication, glycoprotein maturation, assembly, and budding of virions (Delang et al., 2009; Martínez-Gutierrez et al., 2011; Bajimaya et al., 2017; Shrivastava-Ranjan et al., 2018; Episcopio et al., 2019). This effect could be explained because the late stages after viral entry of enveloped viruses also depend on cholesterol biosynthetic pathways (Schmidt et al., 2020). Specifically, it was reported that ATV can affect the virus-induced formation of lipid droplets (LD; Episcopio et al., 2019). Recently, these cell organelles have been proposed as hubs for SARS-CoV-2 genome replication and viral particle assembly (da Silva Gomes Dias et al., 2020; Pagliari et al., 2020). According to these antecedents, atorvastatin treatment could affect the replication and assembly stages of SARS-CoV-2 by blocking LD production. The results presented in this article also showed a reduction of the intracellular D614G strain RNA after post-treatment with ATV at 31.2 and 15.6 μM. These results are correlated with previous studies that reported that statins could inhibit the replication stage by modulating host pathways (Episcopio et al., 2019). Conversely, Martínez-Gutierrez et al. reported that a statin (Lovastatin) caused the accumulation of DENV RNA and reduced the release of infectious viral particles, indicating that these compounds could affect viral replication and subsequent steps (Martínez-Gutierrez et al., 2011). In accordance with this evidence, our results showed that ATV induces an accumulation of the viral RNA at 7.8 and 3.9 μM, suggesting a possible antiviral mechanism of ATV depending on concentration. The accumulation of viral RNA could be related to the decrease in isoprenoid formation by statin treatment, causing an alteration in the prenylation of host proteins, necessary in the post-translational modulation of viral proteins (Greenwood et al., 2006; Kumar et al., 2020). It has also been suggested by other viral models that statins can induce retention of structural proteins within the ER, interfering with viral assembly or intracellular transport of viral particles (Martínez-Gutierrez et al., 2011). Furthermore, ATV could be altering the envelope cholesterol of the nascent viral particles, affecting their infectivity (Sanders et al., 2020).

On the other hand, a previous study revealed that the antiviral activity of statins against rotavirus was independent of the cell line used (Ding et al., 2021). Consistent with this report, our data showed that ATV also inhibited D614G strain in Caco-2 (EC50 = 7.4 μM), a colorectal epithelial cell line highly permissive for SARS-CoV-2 infection (Uemura et al., 2021; Wurtz et al., 2021). This finding suggested that ATV treatment could modulate intracellular signaling pathways in both Vero E6 and Caco-2, affecting some of the cholesterol-dependent SARS-CoV-2 replicative cycle stages, such as those mentioned above. However, additional studies are required to elucidate the antiviral mechanism of this compound in both cell lines.

Additionally, taking into account that multiple SARS-CoV-2 variants have been reported around the world, we evaluated the antiviral activity of ATV against one variant of concern (Delta) and one variant of interest (Mu), which have been associated with altered fitness in terms of virulence, transmission, and evasion of host immune response (Khateeb et al., 2021; Otto et al., 2021). Similar to that was reported for IAV (Haidari et al., 2007), our results indicated that ATV had an independent-variant antiviral effect, with EC50 values of 16.8 and 21.1 μM for the Delta and Mu variants, respectively, using Vero E6 cells. These findings are promising because antiviral drugs with broad activity against SARS-CoV-2 variants could potentially help to reduce the chance of progress to severe disease and to solve the problem of the reduced response of variants to vaccines, especially for vulnerable populations, including children, pregnant women, immunocompromised individuals, and people with comorbidities for COVID-19 (Abdelnabi et al., 2021; Acharya et al., 2021).

On the other hand, previous *in silico* studies have suggested a possible effect of statins on the viral particle by interacting with several viral proteins involved in the later stages of the SARS-CoV-2 replicative cycle (Baby et al., 2020; Reiner et al., 2020; Harisna et al., 2021; Pawlos et al., 2021). According to the above and as a complementary of *in vitro* results, this study evaluated *in silico* the interaction of ATV with RdRp and 3CL protease of SARS-CoV-2, which are involved in the viral RNA replication (Calligari et al., 2020) and polyproteins processing, respectively (Reiner et al., 2020). Our results showed a high binding affinity of ATV with 3CL protease (−7.5 kcal/mol) and the active site of RdRp (−6.7 kcal/mol). These binding energies were even equal to or higher than previously reported candidates for COVID-19 treatment (Kumar et al., 2020; Naik et al., 2020). As shown in Figure 7, complexes between ATV and viral proteins were stabilized by hydrogen bonds, hydrophobic, and electrostatic interactions such as π-alkyl, π-Sulfur, and π-cation. According to our findings and the reported evidence (Reiner et al., 2020; Pawlos et al., 2021), it could be suggested that ATV, in addition to exerting an effect on the cholesterol-dependent cellular metabolic pathways that are necessary for the SARS-CoV-2 replicative cycle, is possible that interacts with viral proteins such as RdRp and 3CL protease. However, *in vitro* and *in vivo* experiments demonstrating these interactions and the biological response are necessary to identify whether this compound exerts a direct antiviral mechanism.

Heparin and Chloroquine were used in this study as positive inhibition controls by *in vitro* assays. Heparin was selected as control of the pre-infection treatment because it is a glycosaminoglycan with a strong structural similarity with heparan sulfate, a SARS-CoV-2 coreceptor (Clausen et al., 2020). Heparin inhibited *in vitro* SARS-CoV-2 entry (Tandon et al., 2021) and has shown the ability of binding to RBD of Spike (KD = ~10–11 M; Clausen et al., 2020; Kwon et al., 2020; Mycroft-West et al., 2020), competing with heparan sulfate present on the cell surface and, affecting subsequently, the SARS-CoV-2 adhesion (Clausen et al., 2020; Zhang et al., 2020). Further, this study selected chloroquine as an internal control for pre-post and post-infection treatments. This compound showed *in vitro* antiviral activity against SARS-CoV-2 with EC50 values between 12.8 and 13.5 μM, which were consistent with previous results on Vero E6 cells (EC50 = 1.13–23.9 μM; Wang M. et al., 2020; Conzelmann et al., 2020). Previously, chloroquine has been evaluated through different *in vitro* antiviral assays against SARS-CoV-2, affecting the early and late stages of the viral replicative cycle (Uzunova et al., 2020). It has also been suggested that chloroquine may interfere with the glycosylation of ACE2 receptors and increase the endosomal pH required for the fusion step. It could also affect the post-translational modification of viral proteins and the maturation of the viral particle (Devaux et al., 2020; Wang M. et al., 2020).

In addition, Remdesivir and Chloroquine were selected as controls of the interaction with RdRp and 3CL protease of SARS-CoV-2, respectively, by *in silico* methodology. Remdesivir is a nucleoside analog that inhibited SARS-CoV-2 RdRp by *in vitro* and *in silico* methodologies (Koulgi et al., 2020; Kokic et al., 2021). On the other hand, chloroquine was identified as a SARS-CoV-2 protease inhibitor (Ki = 0.56 μM) by continuous kinetic assays (Li et al., 2020) and showed a favorable binding affinity with 3CL protease, using bioinformatical methods (Nimgampalle et al., 2020). According to the expected results, favorable binding affinities were obtained between viral proteins and selected positive controls (Table 3).

The concentrations in which ATV demonstrated antiviral potential were similar to those previously reported for other viruses such as the Ebola virus (EBOV; Shrivastava-Ranjan et al., 2018), DENV (Bryan-Marrugo et al., 2016), and ZIKV (Españo et al., 2019). Although the ATV concentrations used *in vitro* are not comparable with the doses applied in humans (Björkhem-Bergman et al., 2011), this work allows us to make an approximation to the benefits of this statin for the treatment of SARS-CoV-2; however, it is necessary to validate its antiviral effect in human cell lines (Li et al., 2020) and particularly, in a non-human animal model, in which the concentrations of the compound can be better adjusted to those of therapeutic use in humans (Minz et al., 2020).

Previous studies reported the multiple effects of statins, such as anti-inflammatory, antioxidant, and immunomodulatory effects (Castiglione et al., 2020). Following the above, it would be pertinent to *in vitro* evaluate the anti-inflammatory activity of ATV in SARS-CoV-2 infected peripheral blood mononuclear cells. Besides, given that statins are used for the treatment of dyslipidemias and are associated with a decrease in cardiovascular complications, it would be interesting to know and compare the benefits of ATV treatment in COVID-19 patients with and without a history of hypertriglyceridemia, hypercholesterolemia, cardiovascular diseases, or its risk factors (smoking, diabetes, and obesity), since they have previously been associated with increased severity and death in patients with COVID-19 (Choi et al., 2020; Ganjali et al., 2020; Matsushita et al., 2020; Sabatino et al., 2020). In addition, statins have shown to be beneficial as add-on therapy in patients with different autoimmune inflammatory diseases such as systemic lupus erythematosus, rheumatoid arthritis, and multiple sclerosis (Castiglione et al., 2020). Based on the above and our results, it would be pertinent to evaluate the effect of ATV in patients diagnosed with COVID-19 that had a history of autoimmune diseases.

ATV is a compound widely available, inexpensive, and safe. Furthermore, as it is a second-use drug, its evaluation as a treatment for COVID-19 would imply a shorter duration and cost (Rudrapal et al., 2020). Currently, some studies have shown that statin therapy is associated with a 30% reduction in fatal or severe COVID-19, a lower likelihood of admission to the intensive care unit (Kow and Hasan, 2020; Tan et al., 2020), and an increased chance of asymptomatic infection (Tan et al., 2020). However, more studies are needed to determine the *in vitro* and *in vivo* biological properties of ATV against SARS-CoV-2 infection, although the available evidence suggests that it could be an effective treatment.

## Conclusion

Atorvastatin demonstrated *in vitro* antiviral activity against the ancestral SARS-CoV-2 D614G strain and two emerging variants (Delta and Mu), with an independent effect of the cell line. Due to low cost, availability, well-established safety and tolerability, and the extensive clinical experience of atorvastatin, it could prove valuable in the treatment of COVID-19.

## Data Availability Statement

All data generated or analyzed during this study are included in this published article (and its Supplementary Material).

## Ethics Statement

This study was carried out keeping good records, practicing good data collection and management, transparency of data-sharing, and realistic representation of study results.

## Author Contributions

MTR, WZ-B, AG-S, CG-A, JH-G, and JCH conceived and designed the study and assisted with interpretation of the results and manuscript writing. MZ-C and LF-Á analyzed the data, interpreted the results, and wrote the manuscript. MTR guided and reviewed the research. All authors have contributed to editing this paper; they have approved this final submission.

## Funding

This study was supported by the Universidad de Antioquia (strategy #UdeA responde al COVID-19), CODI (Act 2020-36850), BPIN 2020000100131-SGR, and Universidad Cooperativa de Colombia.

## Conflict of Interest

The authors declare that the research was conducted in the absence of any commercial or financial relationships that could be construed as a potential conflict of interest.

## Publisher’s Note

All claims expressed in this article are solely those of the authors and do not necessarily represent those of their affiliated organizations, or those of the publisher, the editors and the reviewers. Any product that may be evaluated in this article, or claim that may be made by its manufacturer, is not guaranteed or endorsed by the publisher.

## References

[ref1] Abdelnabi R. (2020). Atorvastatin as Adjunctive Therapy in COVID-19 (STATCO19). National Library of Medicine: United States.

[ref2] Abdelnabi R. Foo C. S. de Jonghe S. Maes P. Weynand B. Neyts J. (2021). Molnupiravir inhibits replication of the emerging SARS-CoV-2 variants of concern in a hamster infection model. J. Infect. Dis. 224, 749–753. doi: 10.1093/infdis/jiab361, PMID: 34244768 PMC8408768

[ref3] Acharya A. Pandey K. Thurman M. Klug E. Trivedi J. Sharma K.. (2021). Discovery and evaluation of entry inhibitors for SARS-CoV-2 and its emerging variants. J. Virol. 95, e01437–e01421. doi: 10.1128/JVI.01437-21, PMID: 34550770 PMC8610590

[ref4] Ader F. Bouscambert-Duchamp M. Hites M. Peiffer-Smadja N. Poissy J. Belhadi D.. (2021). Remdesivir plus standard of care versus standard of care alone for the treatment of patients admitted to hospital with COVID-19 (DisCoVeRy): a phase 3, randomised, controlled, open-label trial. Lancet Infect. Dis. 22, 209–221. doi: 10.1016/S1473-3099(21)00485-0, PMID: 34534511 PMC8439621

[ref5] Ahmad J. Ikram S. Ahmad F. Rehman I. U. Mushtaq M. (2020). SARS-CoV-2 RNA dependent RNA polymerase (RdRp) – a drug repurposing study. Heliyon 6:e04502. doi: 10.1016/j.heliyon.2020.e04502, PMID: 32754651 PMC7377705

[ref6] Alexpandi R. de Mesquita J. F. Pandian S. K. Ravi A. V. (2020). Quinolines-based SARS-CoV-2 3CLpro and RdRp inhibitors and spike-RBD-ACE2 inhibitor for drug-repurposing Against COVID-19: an *in silico* analysis. Front. Microbiol. 11:1796. doi: 10.3389/fmicb.2020.01796, PMID: 32793181 PMC7390959

[ref7] Al-Horani R. A. Kar S. Aliter K. F. (2020). Potential anti-COVID-19 therapeutics that block the early stage of the viral life cycle: structures, mechanisms, and clinical trials. Int. J. Mol. Sci. 21:5224. doi: 10.3390/ijms21155224, PMID: 32718020 PMC7432953

[ref8] Baby K.. (2020). Targeting SARS-CoV-2 RNA-dependent RNA polymerase: an *in silico* drug repurposing for COVID-19. F1000Res. 9:1166. doi: 10.12688/f1000research.26359.1, PMID: 33204411 PMC7610171

[ref9] Bajimaya S. Hayashi T. Frankl T. Bryk P. Ward B. Takimoto T. (2017). Cholesterol reducing agents inhibit assembly of type I parainfluenza viruses. Virology 501, 127–135. doi: 10.1016/j.virol.2016.11.011, PMID: 27915128 PMC5201439

[ref10] Berman H. M. Westbrook J. Feng Z. Gilliland G. Bhat T. N. Weissig H.. (2000). The Protein Data Bank. Nucleic Acids Res. 28, 235–242. doi: 10.1093/nar/28.1.235, PMID: 10592235 PMC102472

[ref11] BIOVIA (2020), D.S. Discovery Studio Visualizer Software, Version 16.1 2017. Available at: https://discover.3ds.com/discovery-studio-visualizer-download [Aceesed December, 2020].

[ref12] Björkhem-Bergman L. Lindh J. D. Bergman P. (2011). What is a relevant statin concentration in cell experiments claiming pleiotropic effects? Br. J. Clin. Pharmacol. 72, 164–165. doi: 10.1111/j.1365-2125.2011.03907.x, PMID: 21223360 PMC3141200

[ref13] Blaess M. Kaiser L. Sauer M. Csuk R. Deigner H.-P. (2020). COVID-19/SARS-CoV-2 Infection: Lysosomes and Lysosomotropism Implicate New Treatment Strategies and Personal Risks. Int. J. Mol. Sci. 21:4953. doi: 10.3390/ijms21144953, PMID: 32668803 PMC7404102

[ref14] Boopathi S. Poma A. B. Kolandaivel P. (2020). Novel 2019 coronavirus structure, mechanism of action, antiviral drug promises and rule out against its treatment. J. Biomol. Struct. Dyn. 39, 1–10. doi: 10.1080/07391102.2020.1758788, PMID: 32306836 PMC7196923

[ref15] Bryan-Marrugo O. L. Arellanos-Soto D. Rojas-Martinez A. Barrera-Saldaña H. Ramos-Jimenez J. Vidaltamayo R.. (2016). The anti-dengue virus properties of statins may be associated with alterations in the cellular antiviral profile expression. Mol. Med. Rep. 14, 2155–2163. doi: 10.3892/mmr.2016.5519, PMID: 27431377

[ref16] Calligari P. Bobone S. Ricci G. Bocedi A. (2020). Molecular investigation of SARS-CoV-2 proteins and their interactions with antiviral drugs. Viruses 12:445. doi: 10.3390/v12040445, PMID: 32295237 PMC7232184

[ref17] Castiglione V. Chiriacò M. Emdin M. Taddei S. Vergaro G. (2020). Statin therapy in COVID-19 infection. Eur. Heart J. 6, 258–259. doi: 10.1093/ehjcvp/pvaa042, PMID: 32347925 PMC7197622

[ref18] Choi G. J. Kim H. M. Kang H. (2020). The potential role of dyslipidemia in COVID-19 severity: an umbrella review of systematic reviews. J. Lipid Atheroscler. 9, 435–448. doi: 10.12997/jla.2020.9.3.435, PMID: 33024735 PMC7521969

[ref19] Clausen T. M.., (2020). SARS-CoV-2 infection depends on cellular heparan sulfate and ACE2. bioRxiv [Preprint]. 10.1016/j.cell.2020.09.033 PMC7489987 32970989

[ref20] ClinicalTrials.gov (2020). Intermediate-dose vs standard prophylactic anticoagulation and statin vs placebo in ICU patients with COVID-19 (INSPIRATION). National Library of Medicine: United States.

[ref21] Conzelmann C. Gilg A. Groß R. Schütz D. Preising N. Ständker L.. (2020). An enzyme-based immunodetection assay to quantify SARS-CoV-2 infection. Antivir. Res. 181:104882. doi: 10.1016/j.antiviral.2020.104882, PMID: 32738255 PMC7388004

[ref001] Corman V. M. Landt O. Kaiser M. Molenkamp R. Meijer A. Chu D. K.. (2020). Detection of 2019 novel coronavirus (2019-nCoV) by real-time RT-PCR. Euro Surveill. 25:2000045. doi: 10.2807/1560-7917.ES.2020.25.3.2000045 PMC6988269 31992387

[ref22] da Silva Gomes Dias S. Soares V. C. Ferreira A. C. Sacramento C. Q. Fintelman-Rodrigues N. Temerozo J. R.. (2020). Lipid droplets fuel SARS-CoV-2 replication and production of inflammatory mediators. PLoS Pathog. 16:e1009127. doi: 10.1371/journal.ppat.1009127, PMID: 33326472 PMC7773323

[ref23] Delang L. Paeshuyse J. Vliegen I. Leyssen P. Obeid S. Durantel D.. (2009). Statins potentiate the in vitro anti-hepatitis C virus activity of selective hepatitis C virus inhibitors and delay or prevent resistance development. Hepatology 50, 6–16. doi: 10.1002/hep.22916 19437494

[ref24] Devaux C. A. Rolain J. M. Colson P. Raoult D. (2020). New insights on the antiviral effects of chloroquine against coronavirus: what to expect for COVID-19? Int. J. Antimicrob. Agents 55:105938. doi: 10.1016/j.ijantimicag.2020.105938, PMID: 32171740 PMC7118659

[ref25] Díaz F. J. Aguilar-Jiménez W. Flórez-Álvarez L. Valencia G. Laiton-Donato K. Franco-Muñoz C.. (2020). Aislamiento y caracterización de una cepa temprana de SARS-CoV-2 durante la epidemia de 2020 en Medellín, Colombia. Biomédica 40, 148–158. doi: 10.7705/biomedica.5834, PMID: 33152198 PMC7676823

[ref26] Ding S. Yu B. van Vuuren A. J. (2021). Statins significantly repress rotavirus replication through downregulation of cholesterol synthesis. Gut Microbes 13:1955643. doi: 10.1080/19490976.2021.1955643, PMID: 34369301 PMC8354672

[ref27] Episcopio D. Aminov S. Benjamin S. Germain G. Datan E. Landazuri J.. (2019). Atorvastatin restricts the ability of influenza virus to generate lipid droplets and severely suppresses the replication of the virus. FASEB J. 33, 9516–9525. doi: 10.1096/fj.201900428RR, PMID: 31125254 PMC6662987

[ref28] Esakandari H. Nabi-Afjadi M. Fakkari-Afjadi J. Farahmandian N. Miresmaeili S. M. Bahreini E. (2020). A comprehensive review of COVID-19 characteristics. Biol. Proced. Online 22:19. doi: 10.1186/s12575-020-00128-2, PMID: 32774178 PMC7402395

[ref29] Españo E. Nam J. H. Song E. J. Song D. Lee C. K. Kim J. K. (2019). Lipophilic statins inhibit Zika virus production in Vero cells. Sci. Rep. 9, 11461–11461. doi: 10.1038/s41598-019-47956-1, PMID: 31391514 PMC6685969

[ref30] FDA (2020). Coronavirus (COVID-19) Update: FDA Authorizes Drug Combination for Treatment of COVID-19. Available at: https://www.fda.gov/news-events/press-announcements/coronavirus-covid-19-update-fda-authorizes-drug-combination-treatment-covid-19 (Accessed January, 2021).

[ref31] FDA (2021a). FDA Approves First Treatment for COVID-19. Available at: https://www.fda.gov/news-events/press-announcements/fda-approves-first-treatment-covid-19 (Accessed December, 2021).

[ref32] FDA (2021b). Coronavirus (COVID-19) Update: FDA Authorizes Drug Combination for Treatment of COVID-19. Available at: https://www.fda.gov/news-events/press-announcements/coronavirus-covid-19-update-fda-authorizes-drug-combination-treatment-covid-19

[ref33] Fedson D. S. Opal S. M. Rordam O. M. (2020). Hiding in plain sight: an approach to treating patients with severe COVID-19 Infection. MBio 11, e00398–e00320. doi: 10.1128/mBio.00398-20, PMID: 32198163 PMC7157814

[ref34] Feng B. Xu L. Wang H. Yan X. Xue J. Liu F.. (2011). Atorvastatin exerts its anti-atherosclerotic effects by targeting the receptor for advanced glycation end products. Biochim. Biophys. Acta 1812, 1130–1137. doi: 10.1016/j.bbadis.2011.05.007, PMID: 21651980 PMC3143240

[ref35] Ganjali S. Bianconi V. Penson P. E. Pirro M. Banach M. Watts G. F.. (2020). Commentary: statins, COVID-19, and coronary artery disease: killing two birds with one stone. Metab. Clin. Exp. 113, 154375–154375. doi: 10.1016/j.metabol.2020.154375, PMID: 32976855 PMC7511211

[ref36] Gao Y. Yan L. Huang Y. Liu F. Zhao Y. Cao L.. (2020). Structure of the RNA-dependent RNA polymerase from COVID-19 virus. Science 368, 779–782. doi: 10.1126/science.abb7498, PMID: 32277040 PMC7164392

[ref37] González-Rayasa J. Ana R.-G. José G.-G. José G.-Y. José H.-H. Rosa del Carmen L.-S. (2020). COVID-19 and ACE -inhibitors and angiotensin receptor blockers-: The need to differentiate between early infection and acute lung injury. Rev. Colomb. de Cardiol. 27, 129–131. doi: 10.1016/j.rccar.2020.04.005

[ref38] Greenwood J. Steinman L. Zamvil S. S. (2006). Statin therapy and autoimmune disease: from protein prenylation to immunomodulation. Nat. Rev. Immunol. 6, 358–370. doi: 10.1038/nri1839, PMID: 16639429 PMC3842637

[ref39] Haidari M. Ali M. Casscells S. W. Madjid M. (2007). Statins block influenza infection by down-regulating rho/rho kinase pathway. Circulation 116, 116–117. doi: 10.1161/circ.116.suppl_16.II_7

[ref40] Hanwell M. D. Curtis D. E. Lonie D. C. Vandermeersch T. Zurek E. Hutchison G. R. (2012). Avogadro: an advanced semantic chemical editor, visualization, and analysis platform. J. Chemother. 4:17. doi: 10.1186/1758-2946-4-17, PMID: 22889332 PMC3542060

[ref41] Harisna A. H. Nurdiansyah R. Syaifie P. H. Nugroho D. W. Saputro K. E. Firdayani. (2021). In silico investigation of potential inhibitors to main protease and spike protein of SARS-CoV-2 in propolis. Biochem. Biophys. Rep. 26:100969. doi: 10.1016/j.bbrep.2021.100969, PMID: 33681482 PMC7914023

[ref42] Isusi E. Aspichueta P. Liza M. Hernández M.´. L. Díaz C. Hernández G.. (2000). Short- and long-term effects of atorvastatin, lovastatin and simvastatin on the cellular metabolism of cholesteryl esters and VLDL secretion in rat hepatocytes. Atherosclerosis 153, 283–294. doi: 10.1016/S0021-9150(00)00407-X, PMID: 11164417

[ref43] Jendele L. Krivak R. Skoda P. Novotny M. Hoksza D. (2019). PrankWeb: a web server for ligand binding site prediction and visualization. Nucleic Acids Res. 47, W345–W349. doi: 10.1093/nar/gkz424, PMID: 31114880 PMC6602436

[ref44] Jones H. M. Fang Z. Sun W. Clark L. H. Stine J. E. Tran A. Q.. (2017). Atorvastatin exhibits anti-tumorigenic and anti-metastatic effects in ovarian cancer in vitro. Am. J. Cancer Res. 7, 2478–2490. PMID: 29312801 PMC5752688

[ref45] Kalil A. C. Patterson T. F. Mehta A. K. Tomashek K. M. Wolfe C. R. Ghazaryan V.. (2020). Baricitinib plus Remdesivir for hospitalized adults with Covid-19. N. Engl. J. Med. 384, 795–807. doi: 10.1056/NEJMoa2031994, PMID: 33306283 PMC7745180

[ref46] Khateeb J. Li Y. Zhang H. (2021). Emerging SARS-CoV-2 variants of concern and potential intervention approaches. Crit. Care 25, 244–244. doi: 10.1186/s13054-021-03662-x, PMID: 34253247 PMC8274962

[ref003] Kim S. S. Peng L. F. Lin W. Choe W.-H. Sakamoto N. Kato N.. (2007). A cell-based, high-throughput screen for small molecule regulators of hepatitis C virus replication. Gastroenterology 132, 311–320. doi: 10.1053/j.gastro.2006.10.032, PMID: 17241881

[ref47] Klein S. Cortese M. Winter S. L. Wachsmuth-Melm M. Neufeldt C. J. Cerikan B.. (2020). SARS-CoV-2 structure and replication characterized by in situ cryo-electron tomography. Nat. Commun. 11:5885. doi: 10.1038/s41467-020-19619-7, PMID: 33208793 PMC7676268

[ref48] Kokic G. Hillen H. S. Tegunov D. Dienemann C. Seitz F. Schmitzova J.. (2021). Mechanism of SARS-CoV-2 polymerase stalling by remdesivir. Nat. Commun. 12:279. doi: 10.1038/s41467-020-20542-0, PMID: 33436624 PMC7804290

[ref49] Korber B. Fischer W. M. Gnanakaran S. Yoon H. Theiler J. Abfalterer W.. (2020). Tracking changes in SARS-CoV-2 spike: evidence that D614G increases infectivity of the COVID-19 virus. Cell 182, 812–827.e19. doi: 10.1016/j.cell.2020.06.043, PMID: 32697968 PMC7332439

[ref50] Koulgi S. Jani V. Uppuladinne M. V. N. Sonavane U. Joshi R. (2020). Remdesivir-bound and ligand-free simulations reveal the probable mechanism of inhibiting the RNA dependent RNA polymerase of severe acute respiratory syndrome coronavirus 2. RSC Adv. 10, 26792–26803. doi: 10.1039/D0RA04743K PMC9055499 35515752

[ref51] Kow C. S. Hasan S. S. (2020). Meta-analysis of effect of statins in patients with COVID-19. Am. J. Cardiol. 134, 153–155. doi: 10.1016/j.amjcard.2020.08.004, PMID: 32891399 PMC7419280

[ref53] Kumar R. Mehta D. Mishra N. Nayak D. Sunil S. (2020). Role of host-mediated post-translational modifications (PTMs) in RNA virus pathogenesis. Int. J. Mol. Sci. 22:323. doi: 10.3390/ijms22010323, PMID: 33396899 PMC7796338

[ref54] Kumar Y. Singh H. Patel C. N. (2020). In silico prediction of potential inhibitors for the main protease of SARS-CoV-2 using molecular docking and dynamics simulation based drug-repurposing. J. Infect. Public Health 13, 1210–1223. doi: 10.1016/j.jiph.2020.06.016, PMID: 32561274 PMC7297718

[ref55] Kwon P. S. Oh H. Kwon S. J. Jin W. Zhang F. Fraser K.. (2020). Sulfated polysaccharides effectively inhibit SARS-CoV-2 in vitro. Cell Dis. 6:50. doi: 10.1038/s41421-020-00192-8, PMID: 32714563 PMC7378085

[ref56] Li Z. Li X. Huang Y. Y. Wu Y. Liu R. Zhou L.. (2020). Identify potent SARS-CoV-2 main protease inhibitors via accelerated free energy perturbation-based virtual screening of existing drugs. Proc. Natl. Acad. Sci. 117, 27381–27387. doi: 10.1073/pnas.2010470117, PMID: 33051297 PMC7959488

[ref57] Lotfi M. Hamblin M. R. Rezaei N. (2020). COVID-19: transmission, prevention, and potential therapeutic opportunities. Clin. Chim. Acta 508, 254–266. doi: 10.1016/j.cca.2020.05.044, PMID: 32474009 PMC7256510

[ref58] Lu Y. Liu D. X. Tam J. P. (2008). Lipid rafts are involved in SARS-CoV entry into Vero E6 cells. Biochem. Biophys. Res. Commun. 369, 344–349. doi: 10.1016/j.bbrc.2008.02.023, PMID: 18279660 PMC7092920

[ref002] Marín-Palma D. Tabares-Guevara J. H. Zapata-Cardona M. I. Flórez-Álvarez L. Yepes L. M. Rugeles M. T.. (2021). Curcumin inhibits in vitro SARS-CoV-2 infection in vero E6 cells through multiple antiviral mechanisms. Molecules 26:6900. doi: 10.3390/molecules26226900 34833991 PMC8618354

[ref59] Martínez-Gutierrez M. Castellanos J. E. Gallego-Gómez J. C. (2011). Statins reduce dengue virus production via decreased virion assembly. Intervirology 54, 202–216. doi: 10.1159/000321892, PMID: 21293097

[ref60] Matsushita K. Ding N. Kou M. Hu X. Chen M. Gao Y.. (2020). The relationship of COVID-19 severity with cardiovascular disease and its traditional risk factors: a systematic review and meta-analysis. Glob. Heart 15, 64–64. doi: 10.5334/gh.814, PMID: 33150129 PMC7546112

[ref61] McIver L. A. Siddique S. M.. (2020). Atorvastatin. Available at: https://www.ncbi.nlm.nih.gov/books/NBK430779/ (Aceesed January, 2021).

[ref62] Meechan P.J. Potts J., (2020). Biosafety in Microbiological and Biomedical Laboratories.

[ref63] Mehrbod P. Ideris A. Omar A. R. Hair-Bejo M. (2012). Evaluation of antiviral effect of atorvastatin on H1N1 infection in MDCK cells. Afr. J. Microbiol. Res. 6, 5715–5719. doi: 10.5897/AJMR12.1011

[ref64] Mendonça L. Howe A Gilchrist JB Sun D Knight ML Zanetti-Domingues LC., (2020). SARS-CoV-2 assembly and egress pathway revealed by correlative multi-modal multi-scale Cryo-imaging. bioRxiv [Preprint]. 10.1038/s41467-021-24887-y PMC8324836 34330917

[ref65] Mendoza E. J. Manguiat K. Wood H. Drebot M. (2020). Two Detailed Plaque Assay Protocols for the Quantification of Infectious SARS-CoV-2. Curr. Protoc. Microbiol. 57:ecpmc105. doi: 10.1002/cpmc.105, PMID: 32475066 PMC7300432

[ref66] Minz M. M. Bansal M. Kasliwal R. R. (2020). Statins and SARS-CoV-2 disease: current concepts and possible benefits. Diabetes Metab. Syndr. 14, 2063–2067. doi: 10.1016/j.dsx.2020.10.021, PMID: 33120281 PMC7582042

[ref67] Mukherjee S. Bhattacharyya D. Bhunia A. (2020). Host-membrane interacting interface of the SARS coronavirus envelope protein: immense functional potential of C-terminal domain. Biophys. Chem. 266, 106452–106452. doi: 10.1016/j.bpc.2020.106452, PMID: 32818817 PMC7418743

[ref68] Mycroft-West C. J. Su D. Pagani I. Rudd T. R. Elli S. Gandhi N. S.. (2020). Heparin inhibits cellular invasion by SARS-CoV-2: structural dependence of the interaction of the spike S1 receptor-binding domain with heparin. Thromb. Haemost. 120, 1700–1715. doi: 10.1055/s-0040-1721319, PMID: 33368089 PMC7869224

[ref69] Naik V. R. Munikumar M. Ramakrishna U. Srujana M. Goudar G. Naresh P.. (2020). Remdesivir (GS-5734) as a therapeutic option of 2019-nCOV main protease - *in silico* approach. J. Biomol. Struct. Dyn. 39, 4701–4714. doi: 10.1080/07391102.2020.1781694, PMID: 32568620 PMC7332877

[ref70] Nimgampalle M. Devanathan V. Saxena A. (2020). Screening of Chloroquine, Hydroxychloroquine and its derivatives for their binding affinity to multiple SARS-CoV-2 protein drug targets. J. Biomol. Struct. Dyn. 39, 4949–4961. doi: 10.1080/07391102.2020.1782265, PMID: 32579059 PMC7332874

[ref71] Otto S. P. Day T. Arino J. Colijn C. Dushoff J. Li M.. (2021). The origins and potential future of SARS-CoV-2 variants of concern in the evolving COVID-19 pandemic. Curr. Biol. 31, R918–R929. doi: 10.1016/j.cub.2021.06.049, PMID: 34314723 PMC8220957

[ref72] Pagliari F. Marafioti M. G. Genard G. Candeloro P. Viglietto G. Seco J.. (2020). ssRNA virus and host lipid rearrangements: is there a role for lipid droplets in SARS-CoV-2 infection? Front. Mol. Biosci. 7:578964. doi: 10.3389/fmolb.2020.578964, PMID: 33134318 PMC7579428

[ref73] Pan H. Peto R. Henao-Restrepo A. M. Preziosi M. P. Sathiyamoorthy V. Abdool Karim Q.. (2021). Repurposed antiviral drugs for Covid-19 - interim WHO solidarity trial results. N. Engl. J. Med. 384, 497–511. doi: 10.1056/NEJMoa2023184, PMID: 33264556 PMC7727327

[ref75] Parquet V. Henry M. Wurtz N. Dormoi J. Briolant S. Gil M.. (2010). Atorvastatin as a potential anti-malarial drug: in vitro synergy in combinational therapy with quinine against plasmodium falciparum. Malar. J. 9:139. doi: 10.1186/1475-2875-9-139, PMID: 20497586 PMC2882376

[ref76] Pawlos A. Niedzielski M. Gorzelak-Pabiś P. Broncel M. Woźniak E. (2021). COVID-19: direct and indirect mechanisms of statins. Int. J. Mol. Sci. 22, 4177. doi: 10.3390/ijms22084177, PMID: 33920709 PMC8073792

[ref77] PFIZER (2010). Pfizer announces European Union approval of a new form of lipitor (atorvastatin) for use in children. Available at: https://www.pfizer.com/news/press-release/press-release-detail/pfizer_announces_european_union_approval_of_a_new_form_of_lipitor_atorvastatin_for_use_in_children (Accessed January, 2021).

[ref78] Reiner Ž. Hatamipour M. Banach M. Pirro M. al-Rasadi K. Jamialahmadi T.. (2020). Statins and the COVID-19 main protease: in silico evidence on direct interaction. Arch. Med. Sci. 16, 490–496. doi: 10.5114/aoms.2020.94655, PMID: 32399094 PMC7212226

[ref79] Rudrapal M. Khairnar S. Jadhav A. (2020). Drug Repurposing (DR): An Emerging Approach in Drug Discovery. London: IntechOpen.

[ref80] Sabatino J. de Rosa S. di Salvo G. Indolfi C. (2020). Impact of cardiovascular risk profile on COVID-19 outcome. A meta-analysis. PLoS One 15:e0237131. doi: 10.1371/journal.pone.0237131, PMID: 32797054 PMC7428172

[ref81] Sacco M. D. Ma C. Lagarias P. Gao A. Townsend J. A. Meng X.. (2020). Structure and inhibition of the SARS-CoV-2 main protease reveal strategy for developing dual inhibitors against Mspro and cathepsin L. Sci. Adv. 6:eabe0751. doi: 10.1126/sciadv.abe0751, PMID: 33158912 PMC7725459

[ref82] Sanders D. W.., (2020). SARS-CoV-2 requires cholesterol for viral entry and pathological syncytia formation. bioRxiv [preprint]. 10.7554/eLife.65962 PMC8104966 33890572

[ref83] Schmidt N. M. Wing P. A. C. McKeating J. A. Maini M. K. (2020). Cholesterol-modifying drugs in COVID-19. Oxf. open immunol. 1:iqaa001. doi: 10.1093/oxfimm/iqaa001, PMID: 33047740 PMC7337782

[ref84] Schöning-Stierand K. Diedrich K. Fährrolfes R. Flachsenberg F. Meyder A. Nittinger E.. (2020). ProteinsPlus: interactive analysis of protein–ligand binding interfaces. Nucleic Acids Res. 48, W48–W53. doi: 10.1093/nar/gkaa235, PMID: 32297936 PMC7319454

[ref85] Shrivastava-Ranjan P. Flint M. Bergeron É. McElroy A. K. Chatterjee P. Albariño C. G.. (2018). Statins Suppress Ebola Virus Infectivity by Interfering with Glycoprotein Processing. MBio 9, e00660–e00618. doi: 10.1128/mBio.00660-18, PMID: 29717011 PMC5930306

[ref86] Simabuco F. M. Tamura R. E. Pavan I. C. B. Morale M. G. Ventura A. M. (2021). Molecular mechanisms and pharmacological interventions in the replication cycle of human coronaviruses. Genet. Mol. Biol. 44:e20200212. doi: 10.1590/1678-4685-gmb-2020-0212 PMC7731901 33237152

[ref87] Singh D. D. Han I. Choi E. H. Yadav D. K. (2020). Recent advances in pathophysiology, drug development and future perspectives of SARS-CoV-2. Front. Cell Dev. Biol. 8:580202. doi: 10.3389/fcell.2020.580202, PMID: 33240881 PMC7677140

[ref88] Su H. X. Yao S. Zhao W. F. Li M. J. Liu J. Shang W. J.. (2020). Anti-SARS-CoV-2 activities in vitro of Shuanghuanglian preparations and bioactive ingredients. Acta Pharmacol. Sin. 41, 1167–1177. doi: 10.1038/s41401-020-0483-6, PMID: 32737471 PMC7393338

[ref89] Tan W. Y. T. Young B. E. Lye D. C. Chew D. E. K. Dalan R. (2020). Statin use is associated with lower disease severity in COVID-19 infection. Sci. Rep. 10:17458. doi: 10.1038/s41598-020-74492-0, PMID: 33060704 PMC7562925

[ref90] Tandon R. Sharp J. S. Zhang F. Pomin V. H. Ashpole N. M. Mitra D.. (2021). Effective inhibition of SARS-CoV-2 entry by heparin and enoxaparin derivatives. J. Virol. 95, e01987–e01920. doi: 10.1128/JVI.01987-20, PMID: 33173010 PMC7925120

[ref91] Trott O. Olson A. J. (2010). AutoDock Vina: improving the speed and accuracy of docking with a new scoring function, efficient optimization, and multithreading. J. Comput. Chem. 31, 455–461. doi: 10.1002/jcc.21334, PMID: 19499576 PMC3041641

[ref92] Uemura K. Sasaki M. Sanaki T. Toba S. Takahashi Y. Orba Y.. (2021). MRC5 cells engineered to express ACE2 serve as a model system for the discovery of antivirals targeting SARS-CoV-2. Sci. Rep. 11:5376. doi: 10.1038/s41598-021-84882-7, PMID: 33686154 PMC7940632

[ref93] Uzunova K. Filipova E. Pavlova V. Vekov T. (2020). Insights into antiviral mechanisms of remdesivir, lopinavir/ritonavir and chloroquine/hydroxychloroquine affecting the new SARS-CoV-2. Biomed. Pharmacother. 131:110668. doi: 10.1016/j.biopha.2020.110668, PMID: 32861965 PMC7444940

[ref94] Villareal V. A. Rodgers M. A. Costello D. A. Yang P. L. (2015). Targeting host lipid synthesis and metabolism to inhibit dengue and hepatitis C viruses. Antivir. Res. 124, 110–121. doi: 10.1016/j.antiviral.2015.10.013, PMID: 26526588 PMC4699661

[ref52] V’kovski P. Kratzel A. Steiner S. Stalder H. Thiel V. (2021). Coronavirus biology and replication: implications for SARS-CoV-2. Biomed. Pharmacother. 19, 155–121. doi: 10.1038/s41579-020-00468-6 PMC7592455 33116300

[ref95] Wang M. Cao R. Zhang L. Yang X. Liu J. Xu M.. (2020). Remdesivir and chloroquine effectively inhibit the recently emerged novel coronavirus (2019-nCoV) in vitro. Cell Res. 30, 269–271. doi: 10.1038/s41422-020-0282-0, PMID: 32020029 PMC7054408

[ref97] Wang H. Yuan Z Pavel M. A. Jablonski S. M. Jablonski J. Hobson R.., (2021). The role of high cholesterol in age-related COVID19 lethality. bioRxiv [Preprint]. doi: 10.1101/2020.05.09.086249

[ref98] Wang Y. Zhang D. du G. du R. Zhao J. Jin Y.. (2020). Remdesivir in adults with severe COVID-19: a randomised, double-blind, placebo-controlled, multicentre trial. Lancet 395, 1569–1578. doi: 10.1016/S0140-6736(20)31022-9, PMID: 32423584 PMC7190303

[ref99] WHO (2020a). COVID-19: cronología de la actuación de la OMS. Available at: https://www.who.int/es/news/item/27-04-2020-who-timeline---covid-19 (Accessed January, 2022).

[ref100] WHO (2020b). WHO Coronavirus Disease (COVID-19) Dashboard. Available at: https://covid19.who.int/ (Accessed January, 2021).

[ref101] Wolff G. Melia C. E. Snijder E. J. Bárcena M. (2020). Double-membrane vesicles as platforms for viral replication. Trends Microbiol. 28, 1022–1033. doi: 10.1016/j.tim.2020.05.009, PMID: 32536523 PMC7289118

[ref102] Wösten-van Asperen R. M. Bos A. P. Bem R. A. Dierdorp B. S. Dekker T. van Goor H.. (2013). Imbalance between pulmonary angiotensin-converting enzyme and angiotensin-converting enzyme 2 activity in acute respiratory distress syndrome. Pediatr. Crit. Care Med. 14, e438–e441. doi: 10.1097/PCC.0b013e3182a55735, PMID: 24226567

[ref103] Wurtz N. Penant G. Jardot P. Duclos N. la Scola B. (2021). Culture of SARS-CoV-2 in a panel of laboratory cell lines, permissivity, and differences in growth profile. EJCDEU 40, 477–484. doi: 10.1007/s10096-020-04106-0, PMID: 33389257 PMC7778494

[ref104] Yepes-Perez A. F. Herrera-Calderón O. Oliveros C. A. Flórez-Álvarez L. Zapata-Cardona M. I. Yepes L.. (2021). The Hydroalcoholic extract of Uncaria tomentosa (Cat’s claw) inhibits the infection of severe acute respiratory syndrome coronavirus 2 (SARS-CoV-2) *in vitro*. Evid. Based Complement. Alternat. Med. 2021:6679761. doi: 10.1155/2021/6679761, PMID: 33680061 PMC7929665

[ref105] Yuan S. Chan C. C. Y. Chik K. K. H. Tsang J. O. L. Liang R. Cao J.. (2020). Broad-Spectrum host-based antivirals targeting the interferon and lipogenesis pathways as potential treatment options for the pandemic coronavirus disease 2019 (COVID-19). Viruses 12:628. doi: 10.3390/v12060628, PMID: 32532085 PMC7354423

[ref106] Zapatero-Belinchón F. J.. (2021). Fluvastatin mitigates SARS-CoV-2 infection in human lung cells. iScience 24:103469. doi: 10.1016/j.isci.2021.103469, PMID: 34812415 PMC8599137

[ref107] Zhang Q. Chen C. Z. Swaroop M. Xu M. Wang L. Lee J.. (2020). Heparan sulfate assists SARS-CoV-2 in cell entry and can be targeted by approved drugs in vitro. Cell Dis. 6:80. doi: 10.1038/s41421-020-00222-5, PMID: 33298900 PMC7610239

