# Supplementary material for: Atorvastatin Effectively Inhibits Ancestral and Two Emerging Variants of SARS-CoV-2 in vitro
Source: Front Microbiol. 2022 Mar 18;13:721103. doi: 10.3389/fmicb.2022.721103 (PMC8972052; doi:10.3389/fmicb.2022.721103)
Supplement: Supplementary file 1 [file Table_1.DOCX]

Supplementary Material

# Supplementary Figures and Tables

## Supplementary Figures


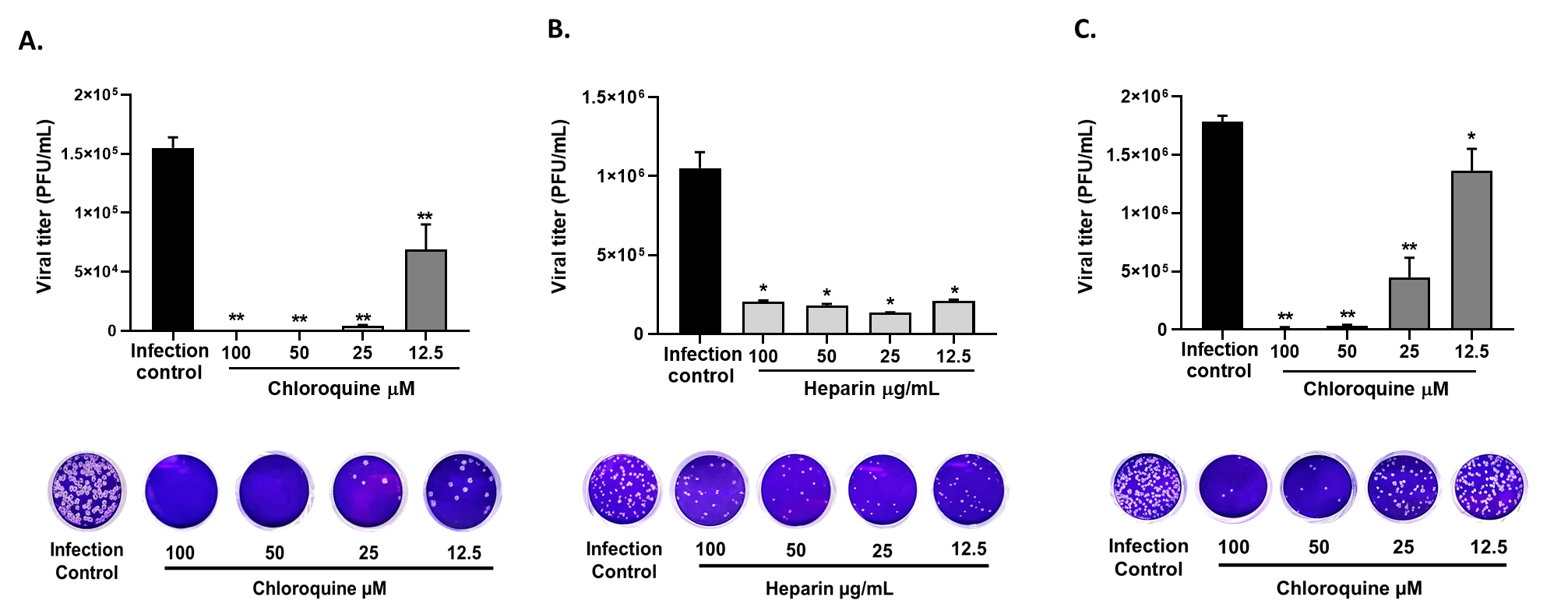


**Supplementary figure 1.** **Antiviral activity of positive inhibition controls against SARS-CoV-2.** The figure represents the viral titer (PFU/mL) of supernatants of Vero E6 after the treatment with positive inhibition controls (n=4). Bars represent mean values ± SEM. *p ≤ 0.05, ** p ≤ 0.01. **A.** Pre-post treatment of CQ. **B.** Pre-infection treatment of Heparin. **C.** Post-infection treatment of CQ. The figures include representative plaques of each treatment.


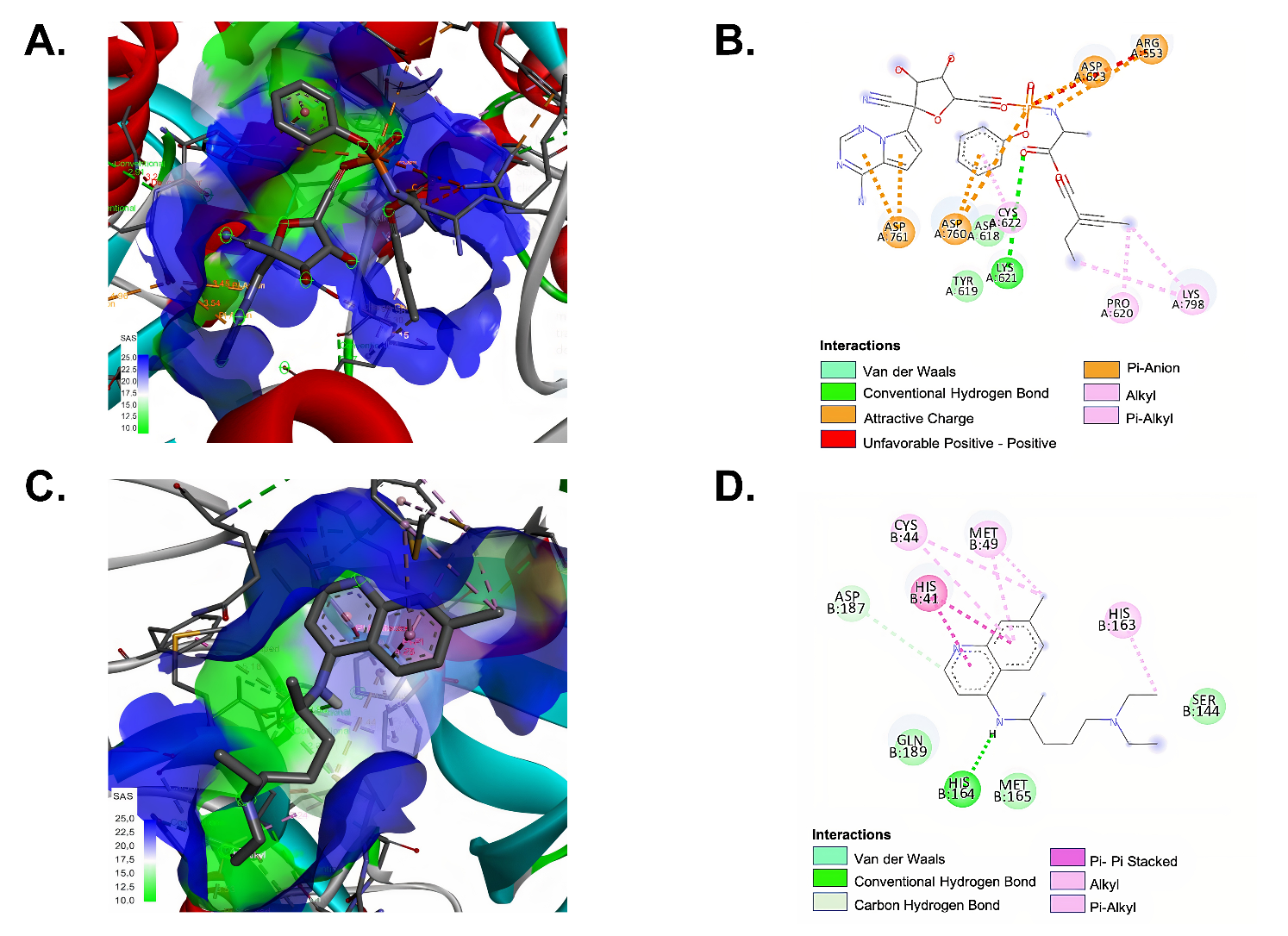


**Supplementary figure 2. Interaction of positive controls with SARS-CoV-2 proteins.** 3D and 2D representations of the main interaction of ATV and two SARS-CoV-2 proteins by molecular docking. The images were obtained using BIOVIA Discovery Studio Visualizer 16.1. Remdesivir interaction with RdRp (PDB:6M71) represented in 3D **(A)** and 2D **(B)** and the complex between Chloroquine and 3CL protease (PDB: 6M2N) of SARS-CoV-2 depicted in 3D **(C)** and 2D **(D).** The interactions formed in the complexes are described in each figure.
